# Remote memory in a Bayesian model of context fear conditioning (BaconREM)

**DOI:** 10.3389/fnbeh.2023.1295969

**Published:** 2024-03-07

**Authors:** Franklin B. Krasne, Michael S. Fanselow

**Affiliations:** ^1^Department of Psychology, University of California, Los Angeles, Los Angeles, CA, United States; ^2^Brain Research Institute, University of California, Los Angeles, Los Angeles, CA, United States; ^3^Department of Psychiatry and Biobehavioral Sciences, University of California, Los Angeles, Los Angeles, CA, United States

**Keywords:** context fear conditioning, remote memory, systems consolidation, hippocampus, neocortex, Bayesian, fear

## Abstract

Here, we propose a model of *remote* memory (BaconREM), which is an extension of a previously published Bayesian model of context fear learning (BACON) that accounts for many aspects of *recently* learned context fear. BaconREM simulates most known phenomenology of remote context fear as studied in rodents and makes new predictions. In particular, it predicts the well-known observation that fear that was conditioned to a recently encoded context becomes hippocampus-independent and shows much-enhanced generalization (“hyper-generalization”) when systems consolidation occurs (i.e., when memory becomes remote). However, the model also predicts that there should be circumstances under which the generalizability of remote fear may not increase or even decrease. It also predicts the established finding that a “reminder” exposure to a feared context can abolish hyper-generalization while at the same time making remote fear again hippocampus-dependent. This observation has in the past been taken to suggest that reminders facilitate access to detail memory that remains *permanently* in the hippocampus even after systems consolidation is complete. However, the present model simulates this result even though it totally moves all the contextual memory that it retains to the neo-cortex when context fear becomes remote.

## 1 Introduction

Context fear conditioning has for some time been a very active area of research. In part, this has been because it is thought that the mechanisms the hippocampus uses to create representations of recently encountered contexts to which fear can then become associated are similar to those it uses to form representations of life experiences during the establishment of episodic memories. Thus, context fear learning provides a convenient laboratory model of certain aspects of episodic memory. The study of context fear learning is also of great interest because of its relevance to understanding and potentially treating pathologies of fear and anxiety.

It is widely believed that the initial creation of contextual fear and episodic memories is the result of very rapidly developed synaptic alterations in the hippocampus and paleo- and neo-cortical circuitry with which the hippocampus interacts, but that over extended periods of time (weeks in rodents and months and years in humans) the information initially stored in the hippocampus-related circuitry becomes recoded and stored in neocortical circuitry through some sort of off-line neural “replay” activity (e.g., Nakashiba et al., 2009; Schwindel and McNaughton, 2011; Buzsáki, 2015; Squire et al., 2015; Pfeiffer, 2020). The content of these largely cortical “systems consolidated” or “remote” memories is thought not to be quite the same as that of the new memories generated at the time of encoding. Whereas new memories are thought to be relatively raw snapshots of the encoded experience or context, allowing recollection of many specific details, systems-consolidated memories are thought to have become integrated into the overall knowledge structure of the individual and to become more generic with many details of the originally experienced context (or event) often, though perhaps not always (as in some highly emotional memories) (e.g., Brown and Kulik, 1977), being lost (e.g., Nadel and Moscovitch, 1997; Moscovitch et al., 2005; Wiltgen and Silva, 2007; Wang et al., 2009; Nadel et al., 2012).

New context fear memories have been studied much more than remote ones, presumably because they are easier to study. However, most of an individual’s memories will ultimately become remote, and it will be remote memories that mostly underlie fear pathologies. So, a full understanding of remote episodic and fear memories is badly needed. Fortunately, information about remote context fear is growing (e.g., Frankland et al., 2004; Frankland and Bontempi, 2005; Wiltgen et al., 2010; Vetere et al., 2011; Kitamura et al., 2017; Tonegawa et al., 2018; Takehara-Nishiuchi, 2021).

The purpose of this study is to extend to remote fear a recent neuro-computational model of context fear learning, BACON (BAyesian CONtext Fear Algorithm) (Krasne et al., 2015), that has been quite successful in explaining and predicting aspects of recently learned context fear and to see how it deals with the increasingly substantial experimental findings on remote context fear memory. BACON and an extension of it that deals specifically with extinction (BaconX) (Krasne et al., 2021) are simplifications of Marr’s Theory of Archicortex (Marr, 1971) and its descendants (e.g., Skaggs and McNaughton, 1992; O’Reilly and McClelland, 1994; Treves and Rolls, 1994; Hasselmo et al., 1996; Hasselmo, 2006; Neunuebel and Knierim, 2014) with an added feature: Representation formation and synaptic strength changes in the hippocampus-cortex circuit, as well as conditioning of fear to these representations in the amygdala, are controlled by Bayesian estimates of how confident an individual can be that the context it recalls really is the one it is currently in. With this addition to Marr’s ideas, BACON can simulate a wide range of context fear phenomenology, including the immediate shock deficit (Fanselow, 1986; Landeira-Fernandez et al., 2006), the gradualness of the onset of expressed fear during exposure to a feared context (Fanselow, 1982; Lester and Fanselow, 1986; Wiltgen et al., 2006; Bae et al., 2015; Lingawi et al., 2018), and false conditioning (Rudy and Oreilly, 1999; Rudy et al., 2020). It has also provided a basis for explaining seeming contradictions in experiments on the effects of DG suppression during encoding and recall (Bernier et al., 2017), and it has provided plausible explanations for a number of non-intuitive effects of conditioning that were carried out at short intervals after placement in a context (Zinn et al., 2020). Moreover, it abolishes the so-called “tradeoff between pattern separation and completion” (O’Reilly and McClelland, 1994; O'Reilly and Rudy, 2001) that arises in the absence of some reasonable mechanism to control when new representations should and should not be created. It has even led to a plausible hypothesis about the basis for known sex differences in context fear conditioning (Trott et al., 2022).

We call the extension of BACON to remote memory BaconREM. Before getting into details, we sketch below the main assumptions we have made and what seem to us to be the most important findings that have followed.

### 1.1 Sketch of assumptions and notable findings

The BACON models all start with a simplified version of Marr’s theory of hippocampal function, which supposes that animals form sparse multicellular hippocampal representations of newly encountered contexts (Marr, 1971; Krasne et al., 2015). The neurons comprising those representations become associated with each other as the result of Hebbian plasticity in the recurrent loops of the hippocampal CA3 region and become associated with cortically represented attributes of the contexts via Hebbian mechanisms at synapses between cortical and hippocampal neurons. Because of these associations, on future occasions observation of a sufficient subset of a context’s attributes activates the full hippocampal representation, which in turn reactivates a cortical version of *all of* the context’s known attributes.

The BACON models then add the assumption that animals evaluate the correctness of the active hippocampal representation by comparing the attributes of the current context that they are observing with the attributes they recall of the active representation; the metric of correctness is the Bayesian weight of evidence (Kass and Rafter, 1995) that they are in fact in the place they remember, which we refer to as “*B_Rep_.”* They then use this evaluation to decide whether the context is a new one, in which case a new representation must be created or is in fact valid, in which case newly observed features of the context can become associated with the existing hippocampal representation (“updating”).

When a hippocampally represented context is fear conditioned, synapses of the representation neurons (or projections of them) on amygdala fear-evoking cells undergo Hebbian potentiation (Fanselow, 1982, 1990, 2000; Young et al., 1994; McDonald, 1998; Fanselow and Gale, 2000; Bauer et al., 2001; Blair et al., 2001; Rudy et al., 2004; Stote and Fanselow, 2004; Zelikowsky et al., 2014), but the extent to which this happens depends on the weight of evidence that the active hippocampal representation really is that of the context the subject is in.

In extending the BACON model to deal with remote memory, we make three main assumptions:

(1) Over time, the hippocampal representations of recently encoded contexts get replaced by cortical versions. Whereas the hippocampal representations are thought to be composed of small sets of hippocampal cells, each of which may also be part of other representations (a “distributed” code), we suppose that their cortical versions are single or small groups of cells that are dedicated to representing just one single context (a “localist” or “non-distributed” code –see Bowers (2009), Riesenhuber and Glezer (2017), and Roy (2017) for discussions relevant to the biological plausibility of localist coding). There is some evidence that the cortical cells that compose the remote version of a contextual representation get selected very soon after the hippocampal representation is created, though their connections to cells representing contextual attributes and to fear-evoking cells develop more slowly during the extended process of systems consolidation (Kitamura et al., 2017), and our model assumes this to be the case. We suppose that cortical remote representation cells, like hippocampal ones, directly or indirectly innervate amygdala fear-evoking cells via Hebbian synapses, but they do so via a different pathway (Tayler et al., 2013; Kitamura et al., 2017); therefore, for recently conditioned fear to become remote, potentiation must be transferred from synapses in the pathway to the amygdala from the hippocampus to ones in the pathway from the cortex.
(2) It is widely believed that systems consolidation depends on hippocampal information sent to the cortex during hippocampal replay events (e.g., Nakashiba et al., 2009; Schwindel and McNaughton, 2011; Buzsáki, 2015; Squire et al., 2015; de Sousa et al., 2019; Pfeiffer, 2020). Consistent with this, though hippocampus-lesioned animals can form some sort of (presumably cortical) contextual representations and be context fear conditioned (Wiltgen et al., 2006; Zelikowsky et al., 2013; Ramanathan et al., 2018) due to some sort of cortical recovery of function (Fanselow, 2010) that becomes operative when hippocampal mechanisms are not available, such conditioned responses never become remote and are fairly rapidly forgotten (Zelikowsky et al., 2012). These facts have led us to construct BaconREM so that *the hippocampus and the information about a context that is encoded within its circuitry are essential for systems consolidation to occur. Furthermore, if new information about a context is learned (“updating”), then the model postulates that the hippocampal circuitry initially stores it just as it does with entirely new learning, and the systems consolidation process is again utilized to make appropriate cortical revisions. However, once a given systems consolidation episode (whether following new learning or updating) is complete, the attribute-hippocampal cell associations and the CA3-CA3 associations that constitute the representation are no longer utilized, and they are erased or over-written so that they do not interfere with new learning*. Although the recurrent collateral and attribute associations of a representation are abandoned, cortical remote representation cells form permanent associations with the CA3 cells of their hippocampal progenitors that make possible generalizations between remotely and recently represented contexts.
(3) It is thought from various perspectives that cortical systems consolidated memories are integrated into an individual’s overall cortical knowledge structure in such a way that the memories are more generic, schematic, or gist-like than hippocampally stored new ones, with details of the original situations to at least some extent being lost (e.g., McClelland et al., 1995, 2020, Nadel and Moscovitch, 1997, Wiltgen and Silva, 2007, Winocur et al., 2007, Wang et al., 2009, Wiltgen et al., 2010, Nadel et al., 2012). An important manifestation of this in the kinds of experiments being considered here is that contextual fear tends to generalize much more broadly after systems consolidation than it did before, a phenomenon that we refer to as “hyper-generalization.” Considering just how the integration of cortical knowledge comes about is beyond the scope of this project. However, we wished our model to incorporate the idea that a given memory has both generic aspects that it shares with other memories and ones specific to particular events or contexts. To do this in as simple a way as possible, we supposed that as an animal experiences many situations, it (somehow) comes to recognize attribute commonalities between them and places them into classes or categories, all of whose members share those common attributes. If a new situation is seen to have enough of the attributes of a known category for that category to be identified, then the individual can assume that the context has all of the category’s known attributes, as well as whatever attributes it has actually observed in the situation. We thus let each of our contexts be in a category and have two kinds of attributes, “Categorical” and “Particular”. Categorical attributes are the same for all contexts in a given category, while Particular attributes are specific to an individual context. Thus, all forests have trees and other common *categorical* attributes, but any given forest has *particular* kinds of trees, terrain, etc. However, all rat testing chambers (at least of a given kind, i.e., in a given *category*) are small boxes without a view of the outside, but *particular* chambers differ in floor textures, odors, lighting, or whatever. If, during the systems consolidation process, BaconREM finds that it knows enough about a context and about the category to which the context belongs to reliably identify the category, all pre-existing knowledge about the category—all of its known categorical attributes—gets added to the set of attributes associated with the context’s cortical representation *even though many of these attributes have never been observed to belong to this specific context*. On the other hand, a number of the attributes that are associated with the context’s hippocampal representation but are not known to be categorical are lost to the cortical version, much as is thought to happen in real animals.

As will be detailed in the Results section, this model emulates a number of well-established experimental findings and makes predictions that, as far as we know, have never been tested. However, we think perhaps the most important insight it has provided is an alternative interpretation for the well-known finding that hyper-generalization of remote fear can be largely abolished, and hippocampal dependence can be re-established by a “reminder” exposure to the feared context. This has been taken to mean that at least some of the contextual details encoded in the hippocampus at the time of original learning remain in the hippocampus even after systems consolidation is complete and that they again become accessible as a result of the reminder stimulus. However, such a conclusion is contrary to the spirit of the Marr-derived theory of hippocampal function on which BACON is based and for which there is much evidence. Since hippocampal contextual representations in Marr-type models, are distributed, a given neuron that is part of one representation may eventually also become part of others; therefore, confusion between one context and another would become rampant if too many contexts were encoded. Thus, it is of critical importance that BaconREM, which postulates that old memories are *always* removed from the hippocampus, simulates the ability of “reminder” exposures to abolish hyper-generalization of remote context fear memories and their return to hippocampal dependence. It does this not because the “reminder” exposures somehow make information still residing in the hippocampus more accessible but rather because they cause new learning (or relearning) about the context’s attributes (updating), which adds information that improves discriminability. There is a return to hippocampal dependence because the new learning must then be integrated with existing cortical knowledge, and this requires information about recently observed attributes that are associated with the hippocampal representation.

## 2 Methods

The present model, BaconREM, which is designed to simulate experiments on remote context fear, is based on the previously published model, BACON (Krasne et al., 2015), which dealt only with recent learning. BaconREM deals with recent learning in the same way as did BACON. Extinction of recent fear, which was treated in a separate model (BaconX, Krasne et al., 2021), was, for simplicity, not incorporated in BaconREM, but the approach would have been the same as that in BaconX, and some expected differences between the extinction of recent and remote context fear are considered in our Discussion section.

### 2.1 Parameters

The values of important BaconREM parameters are listed in Table 1. Not listed are the values of some relatively minor parameters, which are the same as those used in BACON.

**Table 1:** Parameter values and definitions of parameters and abbreviations.

	Brief description	Value
Basic BACON parameters		
*N_ctx_*	Number of representable attributes (kinds of cortical attribute cells)	1 K
*N_atr_*	Number of attributes per context	100
*F*	Number of attribute cells innervating each DG cell	60
*K*	Number of DG & CA3 winners during representation creation and CA3 winners during recall	60
*N_dg_*	Number of DG cells (and of their dedicated CA3 followers)	100 K
*B_new_*	*B_Rep_* below which a new representation gets made	-5
*B_add_*	*B_Rep_* at which newly observed attributes get associated with a representation	15
*B_cnd_*	*B_Rep_* at which conditioning becomes possible (i.e., conditionability is >0)	2
*B_mxCnd_*	*B_Rep_* at which conditionability becomes maximal	12
*B_mxF_*	*B_Rep_* at which fear expression is maximal	4
*α*	Increment in synaptic weight on amygdala cell due to US if conditionability is maximal	1/60
Parameters especially relevant to systems consolidation		
*N_ptc_*	Number of attributes of a context that are particulars	80
*N_cat_*	Number of attributes of a context that are categoricals	20
*N_atr_ = (N_ptc_ + N_cat_)*	Total number of attributes per context	100
*π_ptc_ = (N_ptc_/N_atr_)*	Proportion of attributes of a context that are particulars	0.8
*B_cat_*	*B_Rep_* at which known categorical attributes become associated with a contextual representation	5
*O_catHet_*	Proportion of cat attribute overlap of unrelated contexts in different categories	0.2
*O_ptcHet_*	Proportion of ptc attribute overlap of unrelated contexts in different categories	0.2
*O_ptcHom_*	Proportion of ptc attribute overlap in unrelated contexts in the same category	0.2
*P_oCat_*	Average proportion of attributes known at the time that an established context’s representation became cortical.	0.85
*Z_oRec_*	Average number of attributes of established reps known prior to systems consolidation	85
*κ*	Ceiling on proportion of the *N_ptc_* Unc’s (“unclassified” attributes—i.e., not known to be categoricals) that can get associated with a cortical representation	0.3
*κ_o_*	Ceiling on proportion of Uncs that can get associated with a cortical representation if the context’s category cannot be determined.	0.8
*ϵ*	parameter controlling increase of kappa when a great deal is known about a context	20
*γ*	Factor determining weight of particulars in determining context similarity	0.6
*δ*	Amount by which *B_Rep_* of winner must exceed that of runner-up if new rep is made when winner *B_Rep_* falls below B_new_	5
Additional abbreviations (alphabetical)		
*B_Rep_*	Bayesian weight of evidence for a representation	
*Cnd*	Conditionability of a context (which is a function of *B_Rep_*)	
Cur	A Current attribute	
*Fef*	Fear expression factor	
*O_ptcAB_*	Proportion of overlap of particular attributes of contexts A and B or any other two contexts	
*O_catAB_*	Proportion of overlap of categorical attributes of contexts A and B or any other two contexts	
*O_atrAB_*	Proportion of overlap of all attributes of contexts A and B or any other two contexts	
Rec	A Recalled attribute	
Rep A, B, etc.	Representation A, B, etc.	
*Unc*	An Uncategorized context–one whose category is not known	
*Z_com_*	Number of attributes in common between a set of Current and Recalled attributes	
*Z_cur_*	Number of (Current) attributes so far observed in a session	
*Z_rec_*	Number of attributes of a context that are recalled.	
*Z_o_*	Number of current attributes observed at the time that a representation is created.	

The number of cells listed at the top of the table is approximately 1/100 of rat estimates (see O’Reilly and McClelland, 1994; Treves and Rolls, 1994). An exception is *N_dg_*, which had to be made closer to the biological value (1/10 -th of it) to get biologically plausible results for some simulations. Parameters specifically relevant to systems consolidation, listed separately below the above, were chosen so that the model would simulate known properties of remote context fear as studied in rodents. In particular, the ceiling on the proportion of attributes not known to be categorical (*κ*) must be relatively low, and the proportion of attributes that are particulars (*π_ptc_*) must be substantial to get sufficient hyper-generalization and to simulate the Reminder Effect (see parameter-space graphs at the URL given in the Model Availability section). The overlap of unrelated contexts in the table is approximately equal to expected values for randomly chosen sets of attributes given the number of attributes involved.

### 2.2 Parameters for particular experiments simulated

In all simulations of the Results section, conditioning sessions were 76 computational intervals long, with the US being given at interval 75 (as explained in the Results section, a computational interval occurs whenever a new attribute of the BaconREM’s current context is observed). The duration of all test sessions was 95 intervals.

Except for the simulation in which generalization to a context in a different category than that of the conditioned one was tested, *O_catAB_* = 1. Categories were made different by letting *O_catAB_* = 0.2.

For most of the simulations, the proportion of overlap of particular attributes is *O_ptcAB_* = 0.6. However, for the simulation just mentioned, *O_ptcAB_* was set equal to 0.75 so that the *hypo*-generalization effect would be obvious. For the simulation of the same figure in which a long pre-exposure was given to the to-be-conditioned context, *O_ptcAB_* = 0.8 so that the generalization in the recent case would not be negligible.

Pre-exposure to context B in the simulation showing failure of hyper-generalization when the test context is familiar (Simulation I) was 76 intervals. In the simulation that was designed to show that hyper-generalization fails to occur when the conditioned context is extremely well-known, pre-exposure to context B was 98 intervals.

### 2.3 Evaluating the degree of confidence in an active representation’s correctness (or “validity”)

As explained in the Results section, the mode of operation of the model during each computational interval is determined by the degree of confidence in the currently active representation’s correctness. The degree of confidence in a representation’s validity is indexed by the Bayesian weight of evidence (Kass and Rafter, 1995; Krasne et al., 2015), which we refer to as “*B_Rep_*.” Suppose that after having observed a number *“Z_cur_”* of a current context’s attributes, BaconREM were to activate a representation associated with a number “*Z_rec_*” of recalled attributes and that there was a number *“Z_com_*” of matches between the recalled and current set. Then, BaconREM would compute the probability of getting this number of matches if the recalled context was in fact the current one and also if the current context was just some random place and then calculate the log of the ratio of these probabilities. This is the Bayesian weight of evidence (*B_Rep_*) that the recalled and current contexts are the same. If *B_Rep_* is large and positive, BaconREM probably really is in the recalled context; if it is large and negative, it is probably somewhere else, and if *B_Rep_* is near zero, there was not enough information to make a good decision. It should be noted that the greater *Z_cur_* or *Z_rec_*, the greater will be *B_Rep_* if the representation is valid and the more negative it will be if it is not. Our model postulates that *B_Rep_* is calculated by some extra-hippocampal circuitry (we conjecture pre-frontal). For the simulations of this study, *B_Rep_* was based on expected values of *Z_rec_* and *Z_com_*, given Bacon’s prior experience, assuming that attributes are sampled at random without replacement during a contextual visit.

### 2.4 Model availability

A functional version of BaconREM is available at URL https://www.dropbox.com/scl/fo/edt5unutlzuvr9ghmk88t/h?rlkey=epin8ax8qmsefbr0zjf5zn4jz&dl=0.

## 3 Results

### 3.1 The model

BaconREM is an extension of the BACON model (Krasne et al., 2015), which adds a capacity to develop remote context representations and remote context fear. BaconREM’s treatment of newly created contextual representations, which are hippocampal, and of conditioning to them is the same as in BACON and is incorporated in the following.

#### 3.1.1 New (hippocampal) representations

As in BACON, the attributes of a context are represented cortically by entorhinal cortex-like cells, each of which we think of as coding for a contextual attribute. There are “*N_ctx_*” possible attributes, “*N_atr_*” of which fully characterize a context [note: the number of attributes of each context (*N_atr_*) is one-tenth the total number of representable attributes (*N_ctx_*)] (see Table 1 for values and definitions of all parameters as well as definitions of variable names). In BaconREM, each context belongs, as explained in the Introduction section, to a category, and its attributes are of two types, “categorical,” which are the same for all contexts in a given category, and “particular,” which are specific to a specific context. The *N_atr_* attributes of a context are composed of “*N_cat_*“categorical attributes and “*N_ptc_”* particular ones; for our simulations, we let *N_cat_* be 20 and *N_ptc_* 80. It should be noted that a given attribute might well be categorical for one context but particular for another.

**Figure 1:**
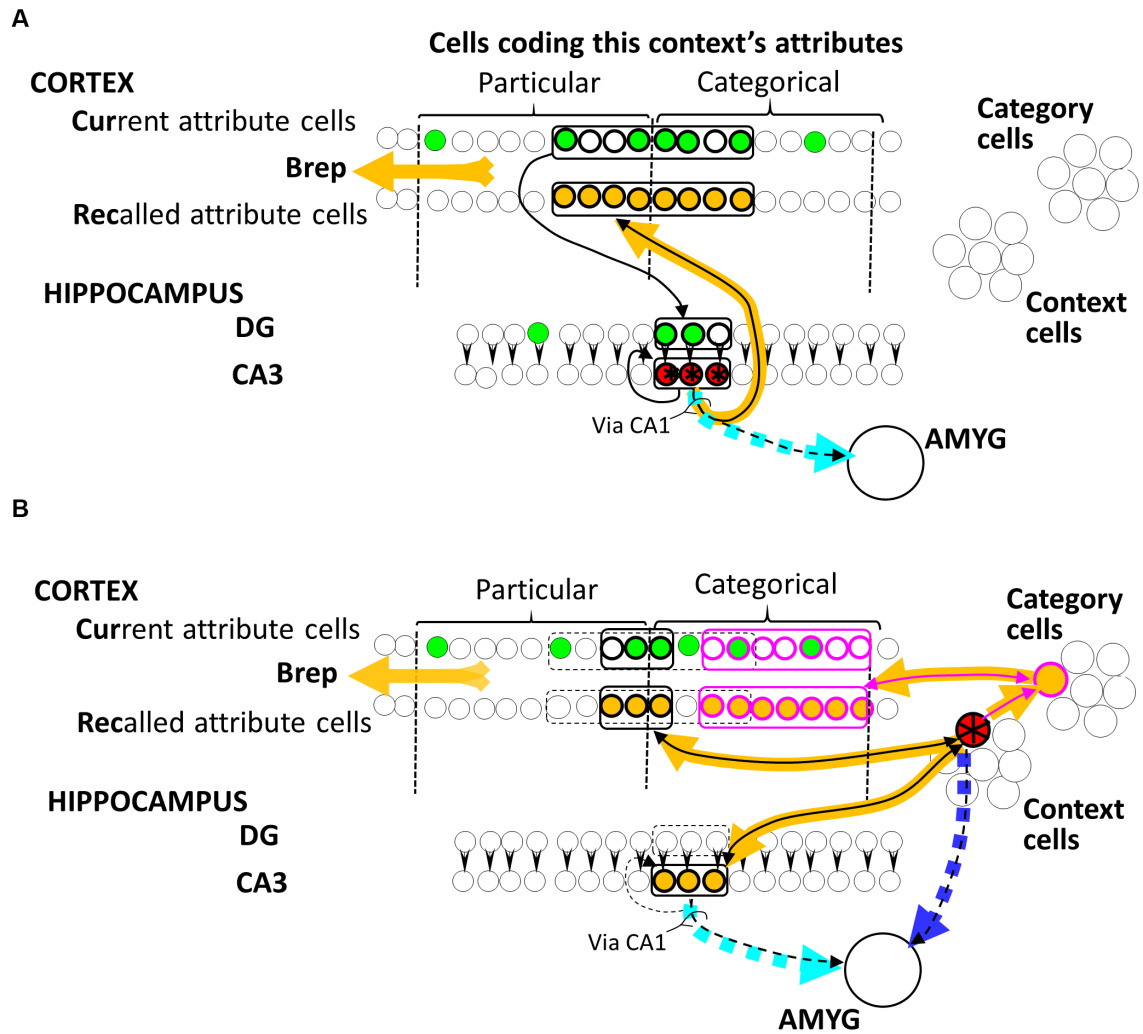
BaconREM representations. Hippocampal **(A)** and Cortical **(B)** representation cells for an illustrative context are colored red and starred. Not indicated in the figure, each Cur cell innervates a random subset of DG cells, while each Rec cell is innervated by all hippocampal CA3 cells, as well as all cortical Context and Category cells, via Hebbian synapses that are ineffective in a naive individual. Amygdala fear-causing cells are innervated by all CA3 cells and all potential Context cells via initially ineffective Hebbian synapses. The particular and current cells representing the attributes of this context are indicated by the brackets at the top of the figure. Synapses that have been made functional by Hebbian potentiation resulting from either representation formation or systems consolidation of this illustrative context are indicated by black arrowheads, with sets of cells making similar connections encircled. Cur and DG cells that might be firing during a visit to the context (depending on what attributes the individual had noticed on this occasion) are green, and cells activated when the red representation cells fire are colored orange. Synapses of the dashed cyan and blue pathways on amygdala cells would be the ones potentiated if a US were to occur when the representation was active. Note that fear conditioned to the hippocampal version would be expressed even if the cortical version were active because of the permanent pathway from the cortical representation to the CA3 cells of its hippocampal progenitor and conversely. “Via CA1” notes that in real animals, this pathway is not directly from CA3; however, CA1 has been omitted in the BACON models because various simplifications made it irrelevant.

Cortical attribute cells come in homologous pairs. One member projects *to* the hippocampus, as illustrated in Figure 1A, and is activated when BaconREM observes the attribute for which that cell codes. We refer to these as “Current” or “Cur” cells (they were called EC_in_ cells in previous articles). The other member of the pair is innervated *by* the hippocampus and is activated when the attribute for which it codes is recalled. We call these “Recalled” or “Rec” cells (these were previously called EC_out_ cells).

As explained in the Introduction section, before systems consolidation, a context’s representation is hippocampal and distributed. Such a representation is portrayed by the red-starred cells in Figure 1A. There is always a fixed number (“*K*”) of cells in a hippocampal representation, which is only a small proportion of the number of cells available to form such representations. The Cur cells that code for attributes project to the dentate gyrus (DG). Each DG cell is innervated by a random subset of Cur cells (of size *“F,”* about half *N_atr_* in number), and it itself innervates a single CA3 cell partner via an innately effective synapse (a simplification of the biological situation where each DG cell innervates a small number of CA3 cells via synapses that are plastic in some way, but not Hebbian, as discussed by Krasne et al., 2015). When a representation is created, the *K DG* cells most richly innervated by the active Cur cells fire and drive their CA3 partners. This leads to the Hebbian potentiation of the synapses between the active Cur and DG cells, between the active CA3 cells and the Rec homologs of active Cur cells, and between one active CA3 cell and another in the recurrent collateral circuitry. Once these potentiations have occurred, the observation of a moderate fraction of the attributes of a familiar context will cause all *K* CA3 cells of its representation to be activated, and thereby, the Rec cells coding for all of the context’s known attributes (which in a real animal would presumably lead to a conscious recollection of the context).

CA3 hippocampal cells also project to the amygdala (a simplification of the biological case where this projection is indirect), where their synapses on fear-evoking cells become potentiated if a CA3 cell is active when a US occurs, thereby causing conditioning.

In rodents, the *K* cells composing a representation have been suggested to be on the order of about half a percent of the set of cells available to form such representations, and this is also the case in BaconREM. Because *K* is such a small proportion of the available cells (i.e., the representation is “sparse”), hippocampal representation overlap is usually very modest even when two contexts have quite similar sets of attributes (i.e., there is considerable “pattern separation” at the level of hippocampal representation cells). The exact degree of overlap will be discussed further when we consider generalization mechanisms.

#### 3.1.2 Remote (cortical) representations

When contextual representations in BaconREM become remote, they are no longer distributed. They are composed of cortical cells that represent just one context. Such a representation is portrayed by the red-starred cell in Figure 1B. Cortical context representation cells, like hippocampal ones, innervate Rec cells, and they also innervate a set of Category cells that innervate and are innervated by Rec cells. As discussed below, categories that are known become represented by a category cell dedicated to representing that category, and the synapses of that cell to and from the Rec cells that represent the category’s known attributes become potentiated. When, during systems consolidation, a context is recognized as belonging to a known category, the synapse of that context’s cortical representation cell on the cell representing that category becomes potentiated so that thereafter, when the context cell fires, it drives the category cell and in turn the Rec cells of all the category’s known attributes. Cortical representation cells also innervate and are innervated by the cells of their hippocampal progenitor. As explained below, these connections serve to allow the generalization of fear between hippocampally and cortically represented contexts. Finally, similar to their hippocampal counterparts, cortical representation cells also innervate the amygdala via Hebbian synapses.

As the result of all these connections, observation of suitable sets of contextual attributes will tend to cause recall of all of the known attributes of a context’s category along with some of its particular attributes and may evoke fear if appropriate conditioning has occurred.

#### 3.1.3 The computational cycle—Decision and Execution

While BaconREM is in a context, it observes (“samples”) the context’s attributes in random order (without replacement), discovering a new one about every half second. The attributes sampled are held in a working memory for the duration of the session. As each new attribute is observed, BaconREM carries out a computational cycle consisting of a Decision phase and an Execution phase.

##### 3.1.3.1 The Decision phase

The purpose of this phase is to activate the representation for which the evidence is best. To do this, the Bayesian weight of evidence (“*B_Rep_*”) is computed (as described in the Methods section) for each of Bacon’s current cortical representations and for whichever hippocampal representation the current set of attributes activates.

**Figure 2:**
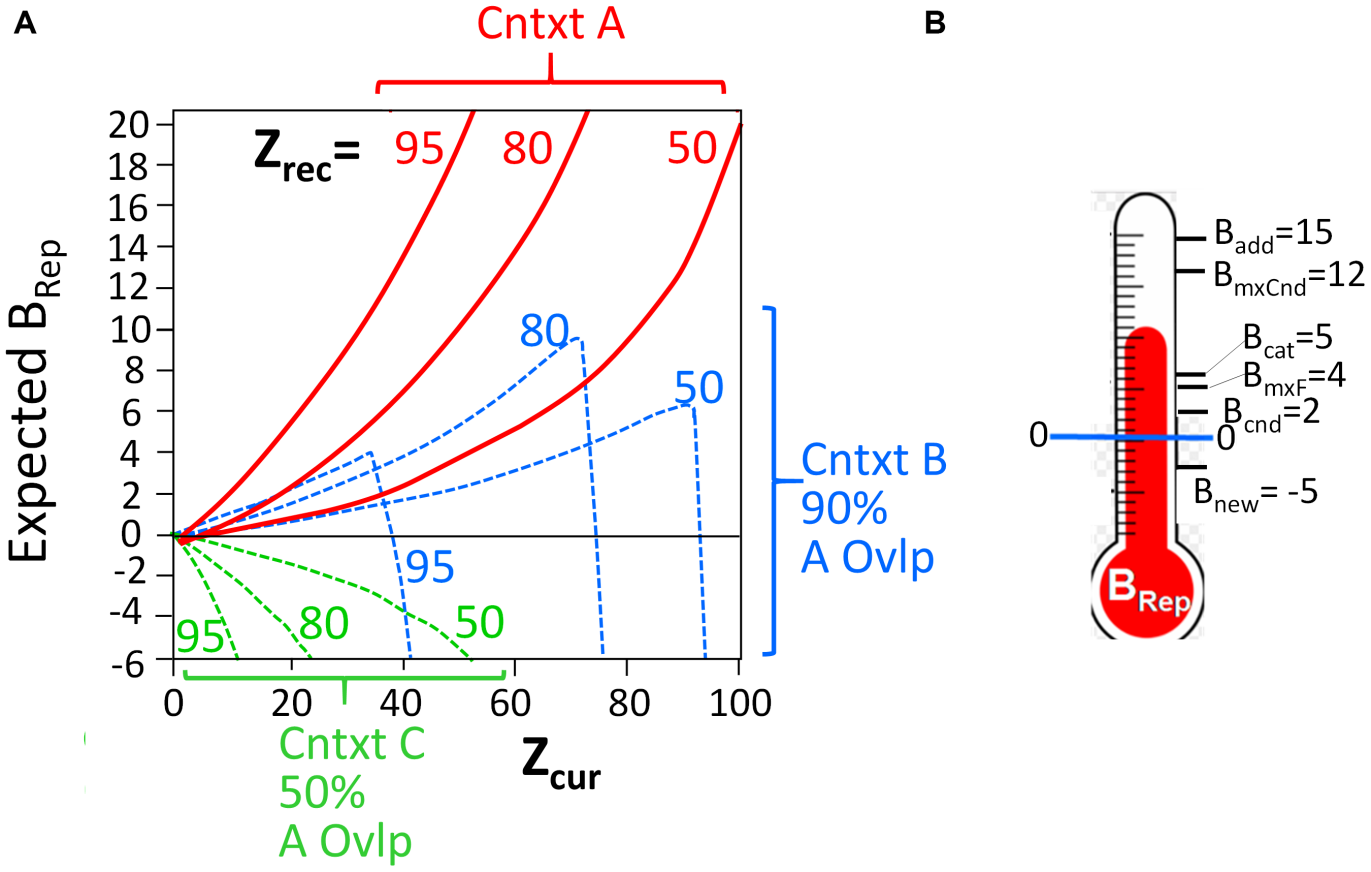
*B_Rep_* as a function of *Z_cur_* and *Z_rec_*, and thresholds for execution. **(A)** Red curves: Expected *B_Rep_* values as a function of *Z_cur_* for three different values of *Z_rec_* when BaconREM is actually in the context it recalls (Context A). Dashed curves: *B_Rep_* values when in a context whose attributes overlap those of the recalled context by 90% (blue) or 50% (green). **(B)** The *B_Rep_* values at which various actions are executed (not to scale).

It should be noted that the computed *B_Rep_* values, which depend on the number of attributes that have been observed (“*Z_cur_”*) and the number associated with the representation (“*Z_rec_*”), are a highly non-linear, and often non-monotonic, function of these variables. Figure 2A portrays the relationship between these variables. The Bayesian mathematics is such that the more one knows about a context, the less sampling of the current context is needed to decide whether it is or is not the hypothesized place, and conversely. It is important to keep in mind that as BaconREM samples contextual attributes in a new context that is similar to, but not the same as, a known one, it can become quite confident that it is in the known place (i.e., very high *B*_*Re*p_) before it “realizes” that this is really somewhere different (i.e., before *B_Rep_* goes very negative). These features are illustrated in the figure and are discussed more fully by Krasne et al. (2015); they lead to some interesting predictions that we will discuss.

##### 3.1.3.2 The execution phase

**Figure 3:**
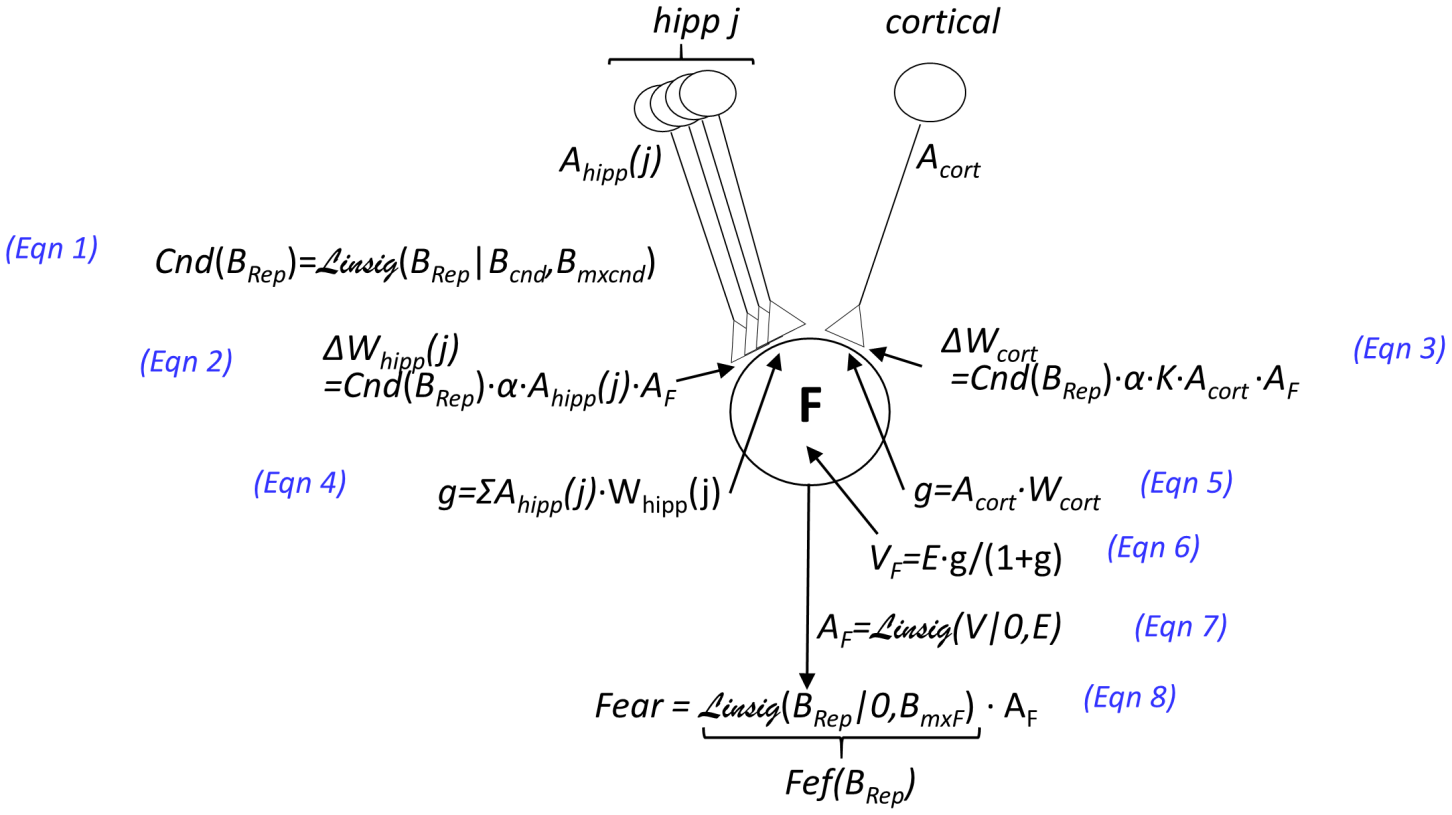
Innervation and plastic properties of amygdala fear-causing cells [inhibitory circuitry responsible for extinction, considered in the BaconX model of Krasne et al. (2021), is omitted here]. Neuron F at the center of the figure is an amygdala fear-causing cell. Activations (i.e., firing rates which are between 0 and a maximum of 1) are denoted by the variable *A*: The activation of the j-th CA3 cell is denoted *A_hipp_*(*j*), that of a cortical context representing cell as *A_cort_*, and that of an amygdala fear causing cell as *A_F_*. The weights of synapses on the amygdala cell are denoted by *W*, and the changes in weights that result from an unconditioned stimulus that depolarizes the amygdala cell are indicated by Δ*W*. The value of the learning rate parameter *α* and other parameters are given in Table 1. The postsynaptic conductances of F cells resulting from representation cell activity are indicated by *g,* the values of which are given as a proportion of the amygdala cell’s leakage conductance. The value of g for the hippocampal input is given by Eqn. 4 and for the cortical input by Eqn. 5. The depolarization *V* resulting from input is given as a function of *g* and the excitatory equilibrium potential *E* (Eq. 6). The depolarization of amygdala cells is converted to a firing rate by the Linsig (“linear sigmoid”) function of *V* and *E* (Eq. 7). The Linsig function, Linsig(*x*|*thrs, mxat*), rises linearly from zero as a function of *x,* starting when *x* = *xthresh* and plateauing at unity when *x = mxat*. The fear that is expressed is equal to the activation of the amygdala cell multiplied by a Fear Expression Factor (*Fef*), which is defined as $Fef\left(B_{\mathrm{Rep}}\right)=\mathrm{Linsig}\left(B_{\mathrm{Rep}}|0,\;B_{mxF}\right)$ .

The winning representation is maximally activated causing several possible, not mutually exclusive actions that depend on the representation’s *B_Rep_* value:

- *A new representation may be created*: If the *B_Rep_* value of the winner is sufficiently negative (specifically, *B_Rep_* is less than the negative parameter *B_new_*) *and* there is no close runner-up (i.e., *B_Rep_* of the winner is an amount *δ* greater than that of the runner-up), then a new representation is created.
- *Conscious recollection of the context may occur*: If the winner is not rejected, the active representation cell activates the Rec cells of all the attributes associated with the representation, presumably causing conscious recollection of the attributes for which they code.
- A US may cause conditioning of fear to the active representation (as spelled out in Figure 3). The extent to which a US will cause conditioning of contextual fear, the “*Conditionability”* (*Cnd*), depends on how confident BaconREM is as to the identity of the context it is in Figure 3, Eq. 1. If the representation is hippocampal, the weight of each of the CA3 cell’s synapses on amygdala fear-evoking cells is increased by an amount *Cnd∙α* (where we refer to *α* as the ‘learning rate parameter”) (Eq. 2), and since the representation consists of *K* CA3 cells, the cumulative weight increase is *K∙Cnd∙α*. If the representation is cortical and composed of only one cell, we assume that conditioning should produce the same excitation of amygdala fear-evoking cells as if it were hippocampal (for a comparable conditionability), and thus, we make the weight increase due to a US the same as the cumulative increase that occurs in the hippocampal case (i.e., *K∙Cnd∙α* – Figure 3, Eq. 3).
- *If fear has previously been conditioned to the winner, its expression depends on B_Rep_*. Expressed fear is proportional to *B_Rep_* with a proportionality constant we call the Fear Expression Factor (Fef) (Eq. 8).
- *The attributes associated with an active representation may be updated during visits to a known context*. If Bacon’s stay in a context has just terminated and Bacon is sufficiently confident that the activated representation was valid (i.e., *B_Rep_* > *B_add_*), then the observed attributes of the known context not already associated with its representation will become so. This is referred to as “updating.”

If the representation is hippocampal, all newly observed attributes will be added to those already associated with the representation (the details of this process are described in Krasne et al., 2015).

If the representation is cortical, then, as detailed below, the hippocampal representation again becomes the active one, and all of the known attributes, including the newly discovered ones and the known attributes of the context’s category (if the category has been identified), become associated with it, and the process of systems consolidation is repeated (note that we do *not* refer to this repetition of the systems consolidation process as “reconsolidation” to avoid confusion with the protein synthesis-dependent “reconsolidation” that is often needed following the recall of previously established learning).

#### 3.1.4 The development of remote representations

The systems consolidation process, which is presumed to operate when the brain is not occupied with current activities, must do a number of things:

(1) *Select the cortical cell(s) that will comprise the representation*. In actuality, the details of this would probably depend on the then-existing cortical knowledge structure and the attributes of the hippocampal representation that is being systems-consolidated. However, we merely assume that representation cells are chosen at random from unused cells of a pre-existing pool designated for that purpose.
(2) *Associate appropriate contextual attributes with the representation*. The attributes of the current context that has become associated with the new hippocampal representation must be compared to the known attributes of known categories, and if a match is found, the matching representation must become associated with the developing cortical representation; this in effect associates the known attributes of the category with the cortical representation. Then, a limited subset of the hippocampal representation’s associated attributes that are not known to be categorical must also become associated with the cortical representation.
(3) *Associate the CA3 cells of the hippocampal representation with the cortical one*. As will be explained when we discuss generalization, mechanisms must be put in place to make possible generalization between recent and remote representations. The approach we use to do this requires that the CA3 cells of a cortical representation’s hippocampal precursor become permanently associated with the cortical representation.
(4) *Transfer fear that has become conditioned to the hippocampal representation to its cortical version*. Fear that was conditioned when the hippocampal representation was active and became associated with it must be transferred to the cortical representation.
(5) *Upgrade the cortical categorical knowledge structure*. The attribute information associated with the context’s hippocampal representation must be used, in conjunction with previously accumulated cortical information, to upgrade the cortical knowledge structure itself. The systems consolidation process presumably attempts to use attribute commonalities between the observed attributes of contexts to try to discover new categories and to add further attributes to existing ones. We do not attempt to model how this is done here, but we assume that it is a potentially time-consuming process that may significantly increase the amount of time needed for the completion of the systems consolidation episode.

**Figure 4:**
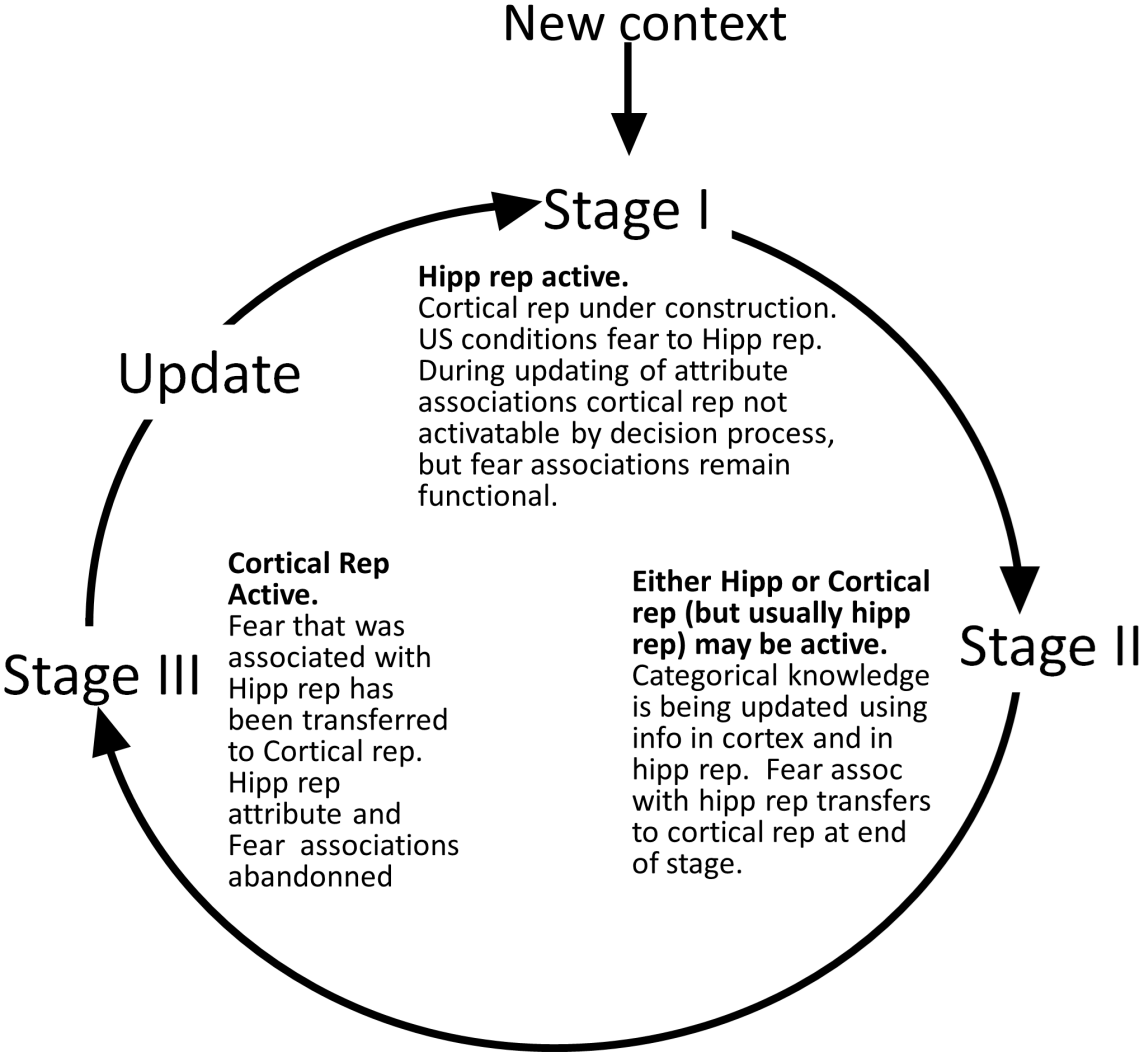
Representation stages. **Stage I**: The selection of a cell that will become the cortical representation (Item 1 of the remote representation development process as described in the text) is thought to be done very soon after the creation of the hippocampal representation (Kitamura et al., 2017). Once a cell has been selected, an appropriate category (if any) must become associated with it (item 2). Doing this is computationally very similar to the necessarily rapid steps carried out during each computational interval, so we suppose it occurs rapidly. Additionally, the CA3 cells of the hippocampal representation become associated with the cortical representation (item 3 above). As explained, when we consider the generalization of fear between contexts with recent and remote representations, these connections between cortical representation cells and the CA3 cells of their hippocampal precursors only function during the execution phases of a computational cycle and so do not influence the outcome of the representation competition of the decision phase. Once a cortical representation cell is chosen, an appropriate category cell, if any, must become associated with it, along with a limited number of attribute cells for attributes that are not known to be part of the context’s category. This process begins by comparing the context’s known attributes with those of each known category and computing for each the weight of evidence that the context belongs to that category. If the *B_Rep_* value for the category whose weight of evidence is greatest exceeds a parameter we call *B_cat_*, that category cell becomes associated with the context representing cell. This in effect associates all the known attributes of the category with the context to which it belongs. Then, a random subset of other attributes associated with the hippocampal representation also becomes associated with the cortical representation. BaconREM does not at this point know whether any given such attribute is categorical or particular, so we refer to them as Unclassified attributes or Unc’s, but given the parameters we have used, they will usually be mostly particulars. The size of the set of Unc’s that gets associated with the developing cortical representation is thought to be small, and we usually cap it to a proportion *κ* of *N_ptc_*, setting *κ* to 0.3 (Table 1) for the simulations done here. However, if the context’s category cannot be determined, we suppose it would be expedient to remember more of its attributes so that it will be more likely to be recognizable in the future and its category more likely to be discoverable at a later time. So, if a category cannot be determined, the ceiling for Unc retention is set to a value *κ_o_*, which is larger than *κ* and taken as 0.8 for all our simulations. Although it is thought that the amount of context-specific information that becomes remote is limited, it is also thought that considerable detail may sometimes be retained, for example, when highly emotional memories become remote (e.g., Brown and Kulik, 1977). We have not incorporated emotion into our model, but it does seem to us that there are some especially familiar contexts for which we hold many details in more or less permanent memory. We, therefore, have designed BaconREM so that when a very great amount has been learned about a particular context, memory for more of its unclassified attributes (which will usually be mostly particulars) is allowed to become remote [we consider in the Discussion section, the possibility that some detail memory might be retained hippocampally rather than cortically]. Figure 5 shows the ceiling that Bacon places on its remote memory of non-categorical attributes as a function of the total amount that is known about the context. For our simulations, we have set the parameter ϵ, which determines how the ceiling on Uncs that becomes remote increases as a function of the total amount known, to 20 (bold curve of Figure 5). The type of circuit that is produced by the systems consolidation process is illustrated in Figure 6B. **Stage II**: Variable, potentially long period during which we hypothesize categories are being revised. **Stage III**: Systems consolidation process has gone as far as it can with available information, and hippocampal representation is abandoned.

We define several stages of systems consolidation (Figure 4) during which the above things are done, as well as an updating procedure that repeats the systems consolidation process to incorporate new attribute information into existing cortical representations.

**Figure 5:**
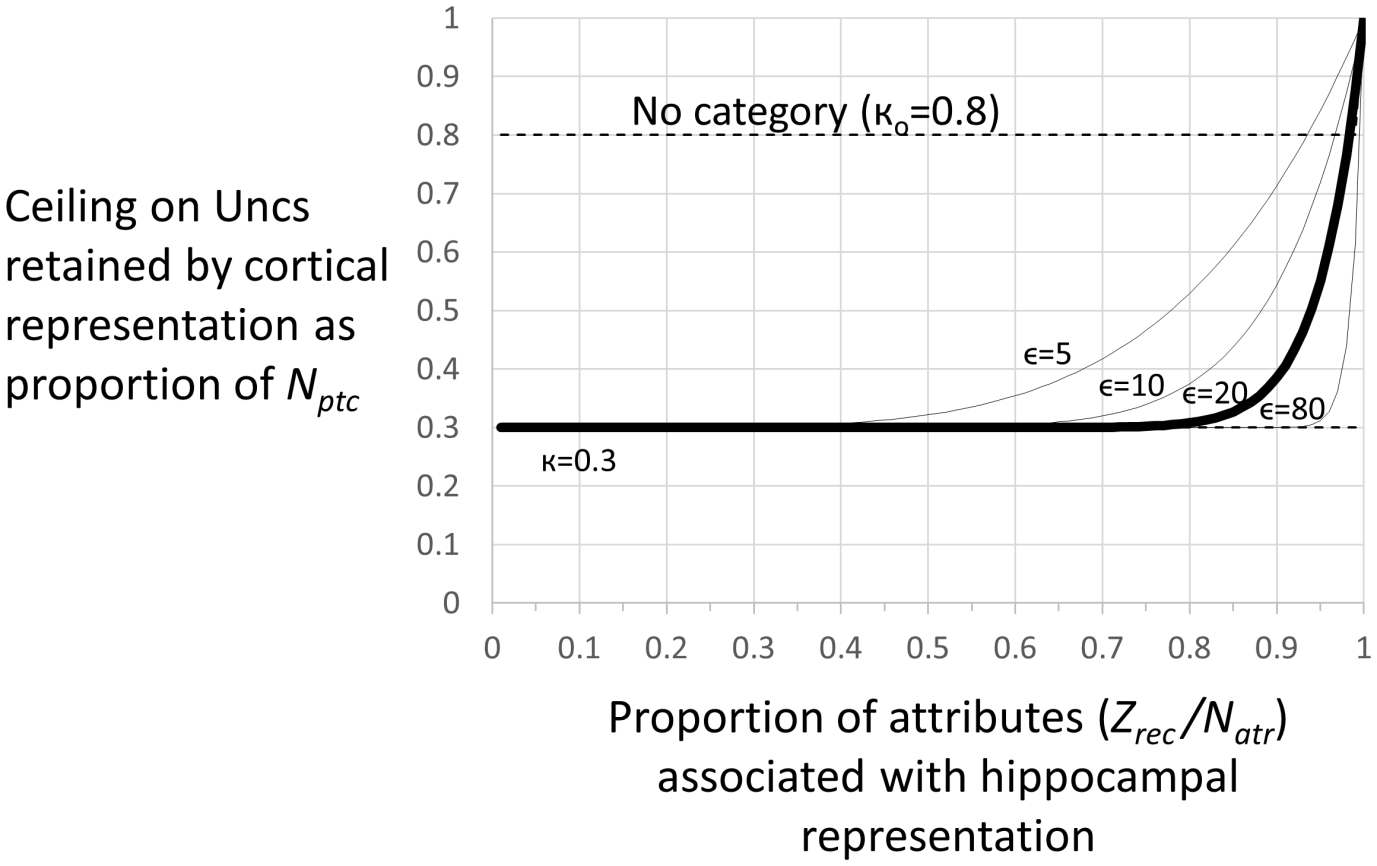
Number of unclassified attributes (“*Unc’*s”) associated with a remote representation. There is a ceiling on the number of (Unc) attributes that are transferred to the cortical version of a representation when the representation becomes remote. This ceiling as a proportion of *N_ptc_* is given by $\mathrm{ceiling}=\kappa +\left(1-\kappa \right)\;.\;{\left(Z_{rec}/N_{atr}\right)}^{\epsilon }$ (Eq. 9) where *Z_rec_* is the number of attributes associated with the hippocampal representation. For the simulations of this study, we have let *κ* = 0.3 and *ϵ* = 20. For these parameters, the ceiling is essentially κ unless a great deal is known about the context. However, if the category of a context cannot be determined, the ceiling is set to a fixed high value к_o_, which we have set to 0.8 for our simulations.

##### 3.1.4.1 Stage I

This is the period that begins directly after the creation of a new hippocampal representation, during which the hippocampal representation is controlling behavior, and the information in it is being used to associate attributes of the context with a cortical representation cell, as detailed in Figure 4.

##### 3.1.4.2 Stage II

Whereas we believe that construction of a cortical representation should be relatively fast, in published experiments on rodents, fear has been found to remain hippocampally dependent and continue to have generalization properties consistent with hippocampal mediation (i.e., does not show the hyper-generalization associated with cortical representations) for a quite variable period ranging from 1 and 2 weeks to well over a month (Kim and Fanselow, 1992; Maren et al., 1997; Anagnostaras et al., 1999; Wiltgen et al., 2010) and sometimes much longer in humans (Reed and Squire, 1998). Thus, there appears to be an extended period after we believe the cortical version of a representation should have been constructed during which the hippocampal representation is still operational and fear conditioned during Stage I is still associated with it. We call this period “Stage II.”

Since contextual fear remains hippocampus-dependent, we presume that even though the cortical representation may be operational, fear that has been conditioned to the hippocampus representation does not actually get copied to it until the end of Stage II. We postulate that during this period, the hippocampal and cortical representations compete for activation but that the hippocampal representation usually wins because the values of *N_cat_* and *N_ptc_* that we have chosen to simulate known findings are such that the hippocampal representation usually has more attribute information than its cortical descendant and thus a higher *B_Rep_* value. Moreover, as discussed below, the model predicts that given these assumptions, there should be circumstances under which the cortical rather than the hippocampal representation wins during Stage II.

We postulate that the hippocampal representation remains intact even after the construction of its cortical descendant so that attribute information associated with it (but not transferred to the cortical version) can contribute to upgrading the cortical categorical knowledge structure itself (item 5 above). We conjecture that animals use commonalities between contexts to try to discover new categories and add further attributes to existing ones. This is computationally *much* more difficult than merely recognizing membership in an existing category, and there is a substantial cognitive science literature concerned with this problem (e.g., Gluck and Bower, 1988; McClelland, 1994; Love et al., 2004; Ashby and Maddox, 2005; Ashby and Maddox, 2011; Carvalho and Goldstone, 2022). Proposing a theory of it and building it into BaconREM is well beyond the scope of this article. We merely suppose that it occurs and may take considerable time; it continues until it has gone as far as it can with available information. We would expect its duration to depend on what old and current information is available and, therefore, to be quite variable.

##### 3.1.4.3 Stage III

When Stage II is finished and fear-producing potentiation has been copied from the hippocampal-amygdala pathway to the cortical representation cell-amygdala pathway, the contextual attribute information of the hippocampal representation is no longer needed for the updating process. We postulate that the representation’s attribute and fear associations, as well as its CA3 recurrent collateral potentiations (but not the associations of the hippocampal representation’s CA3 cells with the cortical representation), are then erased or become subject to being over-written so that the hippocampal circuitry is available to form new representations without interference from old ones (see further below and in the Discussion section). We refer to the post-Stage II period, during which the hippocampal representation is no longer activatable, and its cortical descendant is by default always the one used as Stage III.

##### 3.1.4.4 Fear conditioning and expression in the three stages

Fear becomes conditioned to whichever representation is active at the time of a suitable unconditional stimulus: This will be to the hippocampal representation in Stage I, the cortical representation in Stage III, and whichever version of the representation is active during Stage II.

In order for the fear evoked by the cortical version of a representation to be the same as that produced by its hippocampal progenitor, we postulate that at the Stage II–III transition, the weight of the cortical representation cell synapse on amygdala fear-causing cell(s) is set equal to the cumulative weights of the *K* hippocampal representation cells’ synapses on it. However, when control of behavior is returned to the hippocampal representation as part of revising the associations of an existing cortical representation during its updating, fear previously conditioned to the cortical representation remains so and is expressed via the CA3-cortical representation pathway that mediates generalization between cortical and hippocampally mediated conditional fear as described during our consideration of generalization below. Should conditioning occur when a cortical representation is active, the weight of representation-to-amygdala synapses will be as explained in relation to Figure 3 above.

We presume that the development of potentiation of cortical pathway-amygdala synapses during the transfer of fear from a hippocampal representation to its cortical descendant would be an NMDA-dependent process and, therefore, that pharmacological blocking of NMDA-dependent processes within the amygdala during Stage II would prevent previously learned fear from becoming remote (Table 2, Prediction A), though it would not prevent a cortical representation of the context from replacing the hippocampal one.

**Table 2:** Predictions.

	Prediction	Evidentiary status
A	NMDA receptor block of amygdala during late Stage II abolishes fear during Stage III	Unknown
B	Contexts that are in recognized categories tend to require relatively long exposures for creation of their representations (as indicated by immediate shock deficits), whereas representations of contexts in unknown categories tend to be created relatively rapidly.	Unknown
C	The duration of the immediate shock deficit decreases if BaconREM has been pre-exposed to the context	Fanselow (1986)
D	Contexts with remote representations (Stage III) usually have longer immediate shock deficits and slower fear onsets than contexts having recently created representations	Unknown
E	When a context’s representation becomes remote, fear conditioned to it when its representation was new hyper-generalizes to unfamiliar contexts in the same category	Many examples; e.g., Wiltgen and Silva (2007), Wiltgen et al. (2010), Winocur et al. (2007, 2009)
F	False conditioning is more likely to occur when a conditioned representation is remote than when it is recent	Unknown
G	During Stage II of systems consolidation, BaconREM generalizes as though in Stage I if tested with contexts very similar to the conditioned one and as though in Stage III if tested with more distinct contexts; however, it is always hippocampus-dependent until Stage III. Thus, hyper-generalization to novel contexts may occur well before fear becomes hippocampus-independent if the conditioned and generalization contexts are very different.	Unknown
H	Hyper-generalization of remote fear does not occur if test contexts are familiar.	Unknown
J	Fear conditioned when a representation was recently created *hypo*-generalizes to novel contexts that are in a category different from that of the conditioned context.	Unknown
K	Hyper-generalization of remote fear is much less likely if a conditioned context is extremely well-known.	Biedenkapp and Rudy (2007)
L	Hyper-generalization is abolished and hippocampus-dependence of a remote context fear memory is restored by exposure to the conditioned context (“reminder” effect).	Winocur et al. (2009), Wiltgen and Silva (2007)
M	Reminder effects occur only if exposure to the conditioned context is long enough to generate a *B_Rep_* sufficient to cause updating.	Unknown
N	Pharmacological block of new learning during reminder exposures prevents abolition of hyper-generalization	Unknown

##### 3.1.4.5 Timing of the systems consolidation process

The processing required to associate appropriate attributes with a cortical representation cell is similar to that carried out during each computational cycle, so we would expect Stage I to be relatively brief. However, as said above, we would expect the duration of Stage II to be highly variable and sometimes very lengthy. In fact, as said above, in rodents, the development of hyper-generalization and hippocampus independence, which under the conditions of most experiments indicate the Stage II–III transition, does occur at variable times ranging from as early as 1–2 weeks after representation creation to well over a month. If we assume that the Stage I–II transition usually occurs at a relatively brief and constant time after representation creation, it should occur before the shortest II–III transition and thus at less than 1–2 weeks after representation creation, while the Stage II–III transition occurs at variable times between 1 and 2 weeks and over a month.

#### 3.1.5 Updating a cortical representation

A Stage III representation remains in Stage III indefinitely unless BaconREM is sufficiently confident at the end of a session that an active Stage III representation is valid (i.e., B_Rep_ > B_add_) so that newly observed (or currently re-observed) attributes can become associated with it. In that case, the representation returns to Stage I with all known attributes, including the newly discovered ones, becoming associated with the reconstructed hippocampal representation, and the process of systems consolidation is then repeated to incorporate the new information into the cortical representation and, if possible, to upgrade the categorical knowledge structure.

Such updating of a cortical representation begins by using its knowledge of the CA3 cells of its hippocampal progenitor to re-establish the CA3-CA3 associations of the representation’s hippocampal version so that it will be able to function during Stage I of the systems consolidation process while the cortical version’s attributes are being revised. Then, all known attributes become associated with the re-established hippocampal representation. These include all currently observed attributes and all cortical representation-associated attributes, including those associated with the cortical category representation if it is known. Then, the hippocampally associated attributes become associated with the existing cortical representation during its initial construction. This completes Stage I of the revision process. During Stage II, when both the hippocampal and cortical versions of a representation are operational, any fear that is associated with a cortical representation gets expressed via the hippocampal representation’s CA3 cells’ connections to the cortical representation cell, as explained below when considering generalization.

It should be noted that when, during updating, a limited set of Unclassified (“Unc”) attributes associated with the hippocampal representation is selected to become associated with the representation’s cortical version, the selection, as during *de novo* representation formation, is random. Therefore, while updating a cortical representation may cause new information to be added to the representation, some previously known information may be lost.

In so far as the present theory applies generally to episodic memory, as opposed to just fear conditioning, the updating process could well lead to changes over time in memories of recalled events (Loftus, 2005; Yassa and Reagh, 2013).

Since updating, whether of a hippocampal or a cortical representation, involves new, presumably Hebbian learning, we presume that it would be prevented by NMDA receptor blockers affecting those regions where Hebbian potentiation must occur. We will return to this point, which leads to a crucial test of the model, in the Discussion section.

#### 3.1.6 Generalization

The generalization of context fear has been an important area of study that has shaped ideas about the nature of remote memory. Generalization is a complex affair. Generalization of fear from a conditioned context A to an unconditioned context B might occur, or not, for at least three reasons: (1) A and B might be similar enough that B is simply mistaken for A, especially if B is not already familiar (“misidentification”). Such misidentification is responsible for the hyper-generalization of remote contextual fear. (2) Even if A and B are recognized as being different places or situations, similarities between them might cause a subject to suspect that since A is dangerous, so too might be B. (3) Specific to our model, even if B is mistaken for A (i.e., Rep A is activated in context B), the degree to which the known attributes of A and the observed attributes of B overlap will affect *B_Rep_* and therefore the degree of fear expression.

We will see examples in the simulations described below of all three factors operating. However, we consider now just the situation in which contexts A and B are both known and each activates its appropriate representation.

##### 3.1.6.1 Generalization when one or both representations are hippocampal

**Figure 6:**
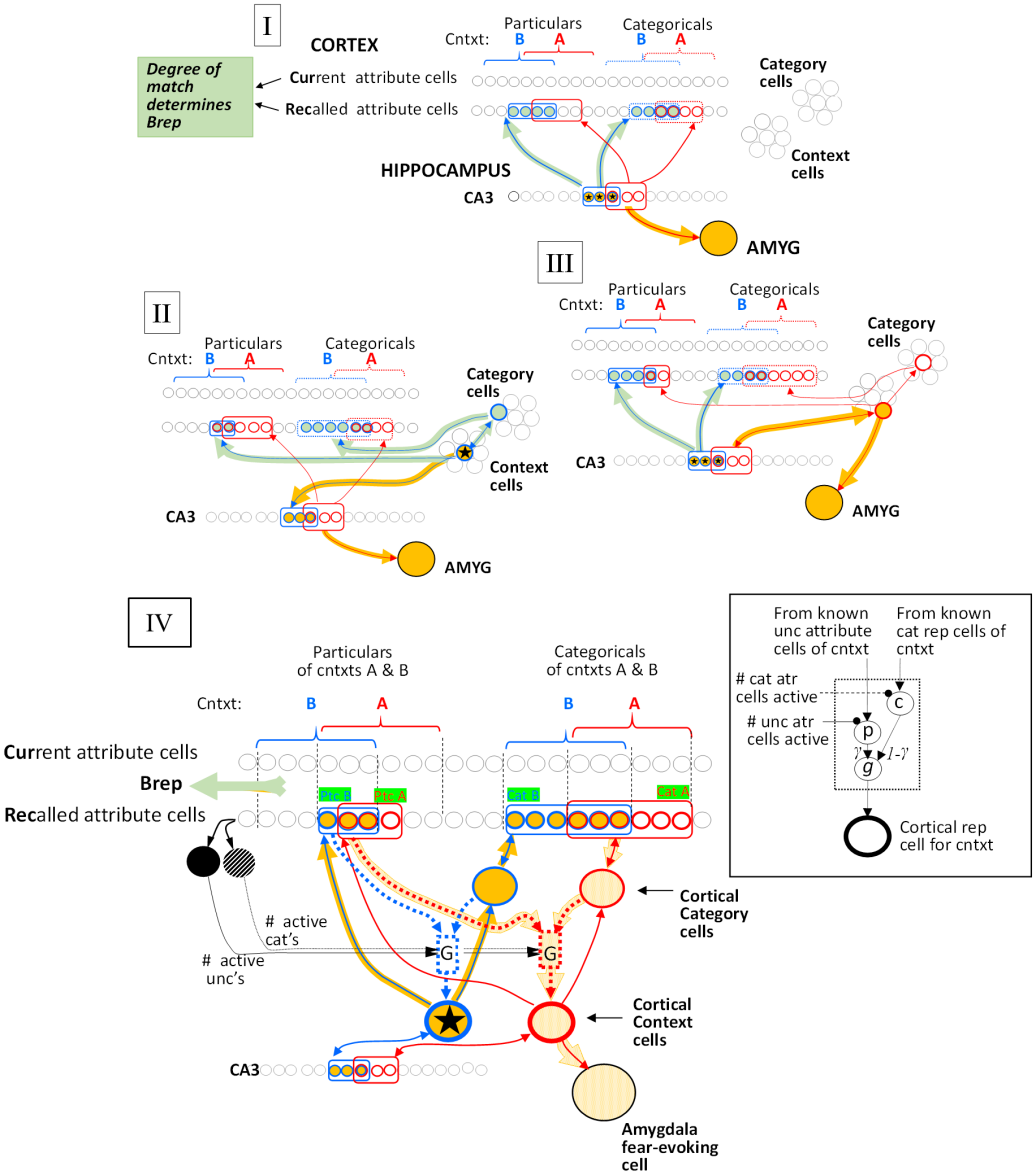
Generalization mechanisms. The red circuitry is that of the conditioned context (context A), and the blue circuitry is that of a test context (B). Active cells and pathways that mediate the generalized fear response are orange, while active cells and pathways that determine *B_Rep_*, which modulates the fear response (see Eq. 8, Figure 3), are gray-green. **(I)** Both context A and B representations are hippocampal. Generalization is due to the overlap of the CA3 representation cells of the two contexts. **(II)** Context A’s representation is hippocampal and Context B’s is cortical. Generalized fear is produced in context B in so far as the CA3 cells of its hippocampal progenitor overlap those of context A’s representation. **(III)** Context A’s representation is cortical and B’s is hippocampal. Generalized fear is produced in B to the extent that the active CA3 cells of B’s representation are in common with the CA3 cells of context A’s hippocampal progenitor. **(IV)** Generalization between cortical representations. Context A, which has been conditioned, is represented by the bold red outlined cortical context cell and Context B, whose representation is active, is represented by the starred blue outlined one. The cells representing the known attributes associated with the context and the category cells of these contexts are outlined with the color corresponding to the representations with which they are associated. Cellular interconnections that have become potentiated due to representation creation or conditioning are shown, but not others. All cortical context cells are innervated by a “G” microcircuit (G for generalization), detailed in the Inset, and portrayed with dotted lines in the main figure. Active cells and pathways are indicated by bold orange markings. The p and c cells of each microcircuit are innervated via potentiated synapses from known Unc cells (mostly particulars) and the context’s cat cell, respectively, and the microcircuit produces an output that is proportional to *G(S_AB_)*. **Details**: The active Context B cell drives its *Z_B,Unc_* known non-categorical attribute cells and (via its category cell) its *Z_A,Cat_* known category attribute cells (indicated by green boxed labels). The expected number of Unc and Cat cells that are common to both A and B are E(Unc’s) = *O_ptcAB_ Z_B,Unc_ Z_AUnc_ / N_ptc_* and E(Cat’s) = *O_catAB_ Z_B,cat_ Z_A,cat_ / N_ptc_*, respectively. During the systems consolidation process, the weights of synapses of known Unc attributes on microcircuit p cells and of known categorical attributes on category cells are normalized so that the sum of their weights are unity. Consequently, the expected excitations due to the active Context A Unc cells synapsing with the microcircuit p cell and the Category attribute Rec cells synapsing with the Cat cell are *O_ptcAB_ Z_B,Unc_* and *O_catAB_ Z_B,cat_*, respectively, and the latter gets passed on to the microcircuit c cell. The microcircuit p and c cells are then subjected to divisive inhibition from the *Z_B,Unc_* and *Z_B,Cat_* active Rec cells, respectively, with the result that the p and c cells excitations, once they are inhibited, are simply *O_ptcAB_* and *O_catAB_*, respectively. Finally, the p and c cells excite the microcircuit output cells (g cells) via synapses of weights *γ* and 1 – *γ,* respectively, so that microcircuit’s expected output is *γ O_ptcAB_ +* (*1 – γ*) *O_catAB_* = *S_AB_*, as desired.

**Figure 7:**
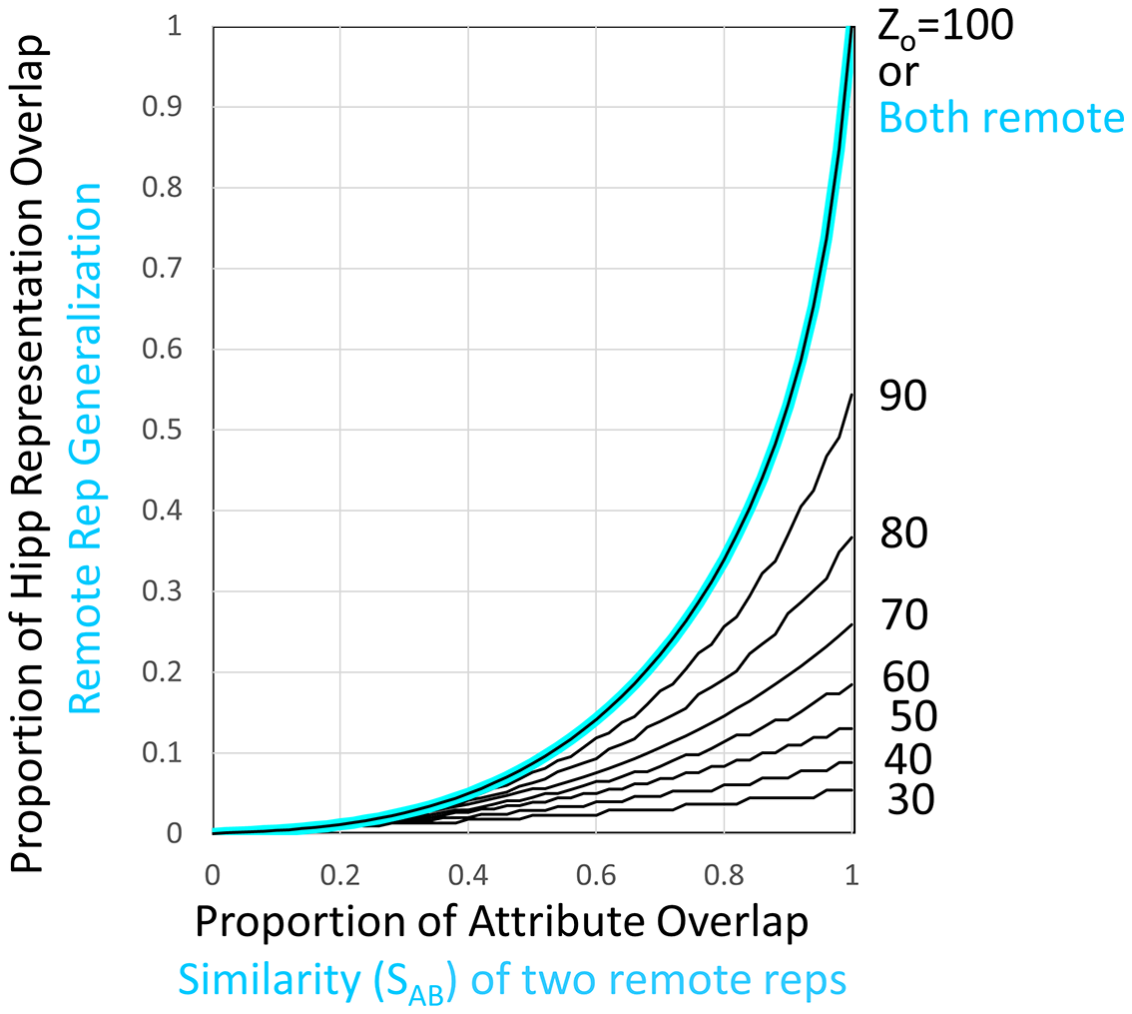
Overlap of hippocampal representations and generalization of two remote representations. Black curves plot hippocampal representation overlap (*O_hippAB_*) as a function of the proportion of attribute overlap (*O_atrAB_*) for various numbers of attributes known. These are based on the assumption that the CA3 cells associated with the *K* most excited dentate cells at the time of representation creation become the hippocampal representation [computed as proposed by O’Reilly and McClelland (1994) and as assumed by Krasne et al. (2015)]. If both representations are cortical, then the generalized response is taken to be the same as the proportion of overlap of hippocampal representations when all *N_atr_* attributes are known, as indicated by the cyan curve and computed by cortical microcircuits of the kind shown in Figure 6.

If context A and B’s representations are both still hippocampal, fear of conditioned context A will be due to potentiation of the synapses of representation A’s CA3 projection onto fear-activating cells of the amygdala, and generalized fear of context B will be produced by those of B’s representation cells that are also are part of A’s representation and hence already have potentiated synapses on fear-activating cells (Figure 6I). As in all Marr-like models, in which representations are composed of the *K* CA3/DG pairs most richly innervated by the set of active Cur cells, the overlap of A and B’s hippocampal representations (“*O_hippAB_*”) will be a very non-linear function of the proportion of overlap of their attributes (“*O_atrAB_*”) and will depend in part on how much was known about the two contexts at the time that their representations were created (“*Z_o_*”), as detailed in Figure 7.

When one pair of representations is hippocampal and the other cortical, generalization occurs via the CA3 cells of a context’s hippocampal representation, which we postulate remains associated with its cortical descendant (Figure 6II,III). These associations only function during the execution phases of a computational cycle, and so do not influence the outcome of the Decision phase’s representation competition.

##### 3.1.6.2 Generalization when both representations are remote

As is clear from Figure 7, when representations are hippocampal, generalization is determined by the overlap of their representations, and as a result, it is rather heavily dependent on how much was known about the contexts at the time of their representation’s creation (i.e., on their *Z_o_* values). This is true even when the contexts are almost identical because unless most of the attributes of a context have been observed by the time a representation is created, different attributes would have been randomly sampled on different occasions even if two contexts were identical; thus, somewhat different hippocampal representations would result. However, when two representations are both remote, we have programmed BaconREM to use more sophisticated cortical mechanisms that allow generalization to be determined in a way that depends only on context similarity and is independent of *Z_o_*. These mechanisms cause generalization between two contexts both of which are remote to depend on a “Similarity” value (“*S_AB_*”), defined as


(10) $$S_{AB}=\gamma \;.\;O_{\mathrm{ptcAB}}+\left(1-\gamma \right)\;.\;O_{\mathrm{catAB}}$$

where *O_ptcAB_* and *O_catAB_* are the proportions of overlap of particular and categorical attributes of contexts A and B, and *γ*, which lies between 0 and 1, determines the relative impact of similarity of categorical versus particular attributes of the two contexts. If a pair of remote contexts are not both categorized, then *S_AB_* is simply the overlap of the totality of their attributes irrespective of categorization status (i.e., *O_atrAB_*).

We then let generalization be equal to G(*S_AB_, Z_o_*) (see Figure 7) for *Z_o_ = N*_*at*r_. This makes the generalization of remote representations be the same as hippocampal ones if all attributes were known, as indicated in the cyan curve of Figure 7. We do this by having each cortical representation cell receive input from an associated microcircuit that causes the representation cell to fire at a rate that depends on *S_AB_*. The relevant circuitry is sketched in Figure 6IV and explained in its legend.

### 3.2 Behavior of the model

In this section, we describe behavior predicted by the model with an emphasis on the kinds of experiments that have already been done and have shaped thinking about the nature of remote context fear memory.

#### 3.2.1 Representation creation and systems consolidation

##### 3.2.1.1 Making new representations and the immediate shock deficit

When BaconREM is first placed in a new context, it will initially activate the representation for whichever of its familiar contexts the evidence is best (i.e., *B_Rep_* is highest). But as it samples more and more of the new context’s attributes, it will eventually ‘realize’ that it is not in fact in the most similar known context (i.e., the favored representation’s *B_Rep_* will fall below *B_new_*), and a new representation will be created. If the attributes of some of the already familiar contexts are similar to those of the new one, as may well be the case, especially for contexts in the same category, it may have to observe a great many of the new context’s attributes before it realizes it is somewhere new, which might require a very long visit to the context.

**Figure 8:**
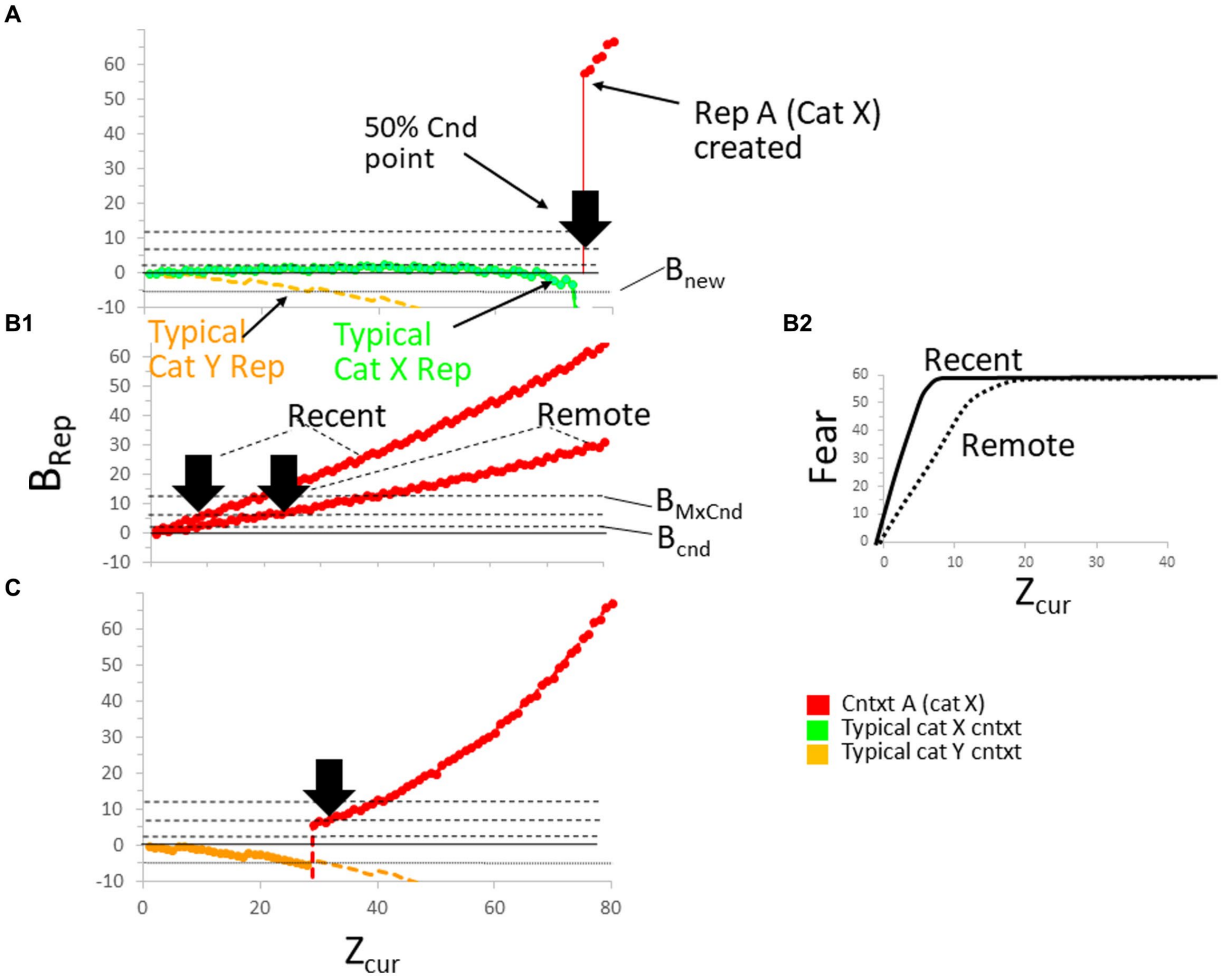
The making and conditioning of representations. **(A)** BaconREM is placed in a new context (Context A) that is in the same category (X) as that of many other contexts with which it is familiar. It takes a long time for Bacon to form a representation of A. Fully explained in the text. **(B**_**1**_**)** Confidence in the correctness of a representation (*B_Rep_*) usually grows more rapidly as a function of *Z_cur_* for a recent (hippocampal) than for a remote (cortical, Stage III) representation because the latter usually has less attributes associated with it. As a result, conditionability increases more rapidly for the recent than for the remote version of a representation. Bold arrowheads indicate the half-maximal conditionability point for the recent and remote versions of Rep A. Inactivating the hippocampus during Stage III before conditioning the remote representation would have no effect. **(B**_**2**_**)** As a result of the more rapid growth of *B_Rep_* of Context A in the recent than remote case, conditioned fear expression increases more rapidly as a function of *Z_cur_* for recent than remote representations. **(C)** The formation of Rep A when BaconREM does not have established representations in its category.

The process of new representation creation is illustrated in Figure 8A for a case in which Bacon is placed in a context A that is new to it (i.e., for which it does not yet have a representation) belonging to a category X for which it *does* have a representation. Since Bacon has a representation of category X, it must have acquired it as the result of considerable prior experience with multiple contexts of that category, and in the graph, we plot the *B_Rep_* value for a typical such context. We also plot the *B_Rep_* value for a typical context of some other category Y; we assume in these calculations that the representations of the typical category X and Y contexts, which we will sometimes refer to as “Established” contexts, were created some time ago and are, therefore, remote. The *B_Rep_* values for the typical X category context are plotted as a green curve and those for the typical Y category context as a yellow one.

The representation with the highest *B_Rep_* value, which will always be the one that gets activated, is indicated in this and other figures by making its *B_Rep_* curve bold. At the start of the session, the typical X context is the one activated because the context into which BaconREM has been put is more similar to it than to the typical Y context because its categorical attributes are the same as those of the unfamiliar context A. At first, Bacon’s confidence grows slightly (i.e., *B_Rep_* increases slightly) as it samples more of A’s attributes, but as it samples still more attributes, *B_Rep_* begins to fall (i.e., it begins to ‘realize’ it is not in the category X context whose *B_Rep_* value we have plotted), and at around *Z_cur_ =* 75, *B_Rep_* falls below *B_new_* (i.e., Bacon ‘realizes’ it is somewhere new), and a representation of context A itself (hereafter “Rep A”) is created.

It will be possible to tell behaviorally when the representation of context A is created by when it becomes conditionable. An unconditional stimulus given before then could in principle cause fear to become conditioned to one of the typical non-A representations, but their *B_Rep_* values would be so low that little or no effective conditioning (which requires *B_Rep_* > *B_cnd_*) would occur. The time it takes for conditioning to become possible is what has been called the “immediate shock deficit” (Fanselow, 1986). We have placed a bold black arrowhead at the point where context A’s conditionability would have reached 50% of its maximum possible value.

The immediate shock deficit in Figure 8A was long because it took a long time for BaconREM to be sure that context A was not in fact one of the category X contexts with which it was already familiar. In Figure 8C, a different specimen of BaconREM was introduced into a new context A, for which it did *not* have a categorical representation. In this case, the BaconREM individual had in the past only experienced contexts in category Y, never once in category X. The *B_Rep_* value for a typical context of category Y is again plotted as a yellow curve. Since new context A has very few attributes in common with the typical category Y context, the context’s weight of evidence is very low or negative. However, since category Y contexts are the only ones BaconREM knows, their *B_Rep_* values are the best ones going, so one of them (in this case, the typical one being considered) is initially activated (as indicated in the graph by making the yellow curve bold). However, as more and more of context A’s attributes are sampled, *B_Rep_* of the category Y contexts will fall below *B_new_*, and a representation of context A gets created, as indicated by the new presence of the bold red context A *B_Rep_* curve. Thus, a representation of context A gets made much sooner when BaconREM is unaware of the category to which it belongs than when it knows many contexts in A’s category and is able to identify that category (Table 2, Prediction B). Thus, representations are more likely to get created for contexts that are in unknown categories than in known ones.

##### 3.2.1.2 The pre-exposure effect

Immediate shock deficits in novel contexts are expected to be fairly long unless animals are placed in contexts very different from any they are familiar with. However, if Bacon is first exposed to a novel context long enough for a representation of the context to be created, and conditioning is then attempted in a later session, the immediate shock deficit will be much shorter, as illustrated in Figure 8B_1_. During the session following that of representation creation, Rep A was activated very soon after the start of the conditioning session, and its *B_Rep_* grew as more and more of the context’s attributes were sampled, reaching *B*_*cn*d_ at around *Z_cur_* = 5, and conditionability became 50% maximal at approximately *Z_cur_* = 10. Many experiments have been done in which it has been shown that the immediate shock deficit is in fact greatly reduced by pre-exposure to a to-be-conditioned novel context (Table 2, Prediction C).

##### 3.2.1.3 Consequences of going remote

When BaconREM’s representation of a new context becomes fully remote (i.e., enters Stage III), it usually loses attribute information, thereby lowering Bacon’s confidence as to its whereabouts and thus reducing both its conditionability and its expression of previously conditioned fear (Table 2, Prediction D). This is illustrated in Figures 6B_1_,B_2_.

#### 3.2.2 Generalization experiments

##### 3.2.2.1 Generalization of fear from a conditioned context to an *unfamiliar* context in the same category

If an animal is conditioned in one context (A) and soon thereafter is tested in a novel context (B) that is not too different, there will usually be some generalization, but fear will be less than in A. However, it is commonly found that if one delays such testing to allow time for systems consolidation to occur, fear of B is almost as great as that of context A (Wiltgen and Silva, 2007; Winocur et al., 2007, 2009; Wiltgen et al., 2010). Such results are generally taken as evidence that the memory of context A has become more generic and knowledge of A’s specific features has been substantially forgotten or become inaccessible; we refer here to this phenomenon as “hyper-generalization.” The onset of hyper-generalization is thought to be correlated with fear becoming hippocampus-independent (Wiltgen et al., 2010).

During tests of generalization in such an experiment, BaconREM, initially lacking a representation of context B, would be expected to at first activate its representation of context A and express considerable fear. But as it observed more and more of context B’s attributes, it would eventually ‘realize’ that it was not in A and create a representation of context B, of which it was not afraid, and thereafter express only an amount of fear commensurate with the overlap of A and B’s pattern-separated representations. Representations of the un-feared context B would be created much sooner during recent than during remote tests because the hippocampal representation would be relatively rich in attributes specific to context A, whereas the cortical one would be relatively rich in categorical attributes common to contexts A and B.

**Figure 9:**
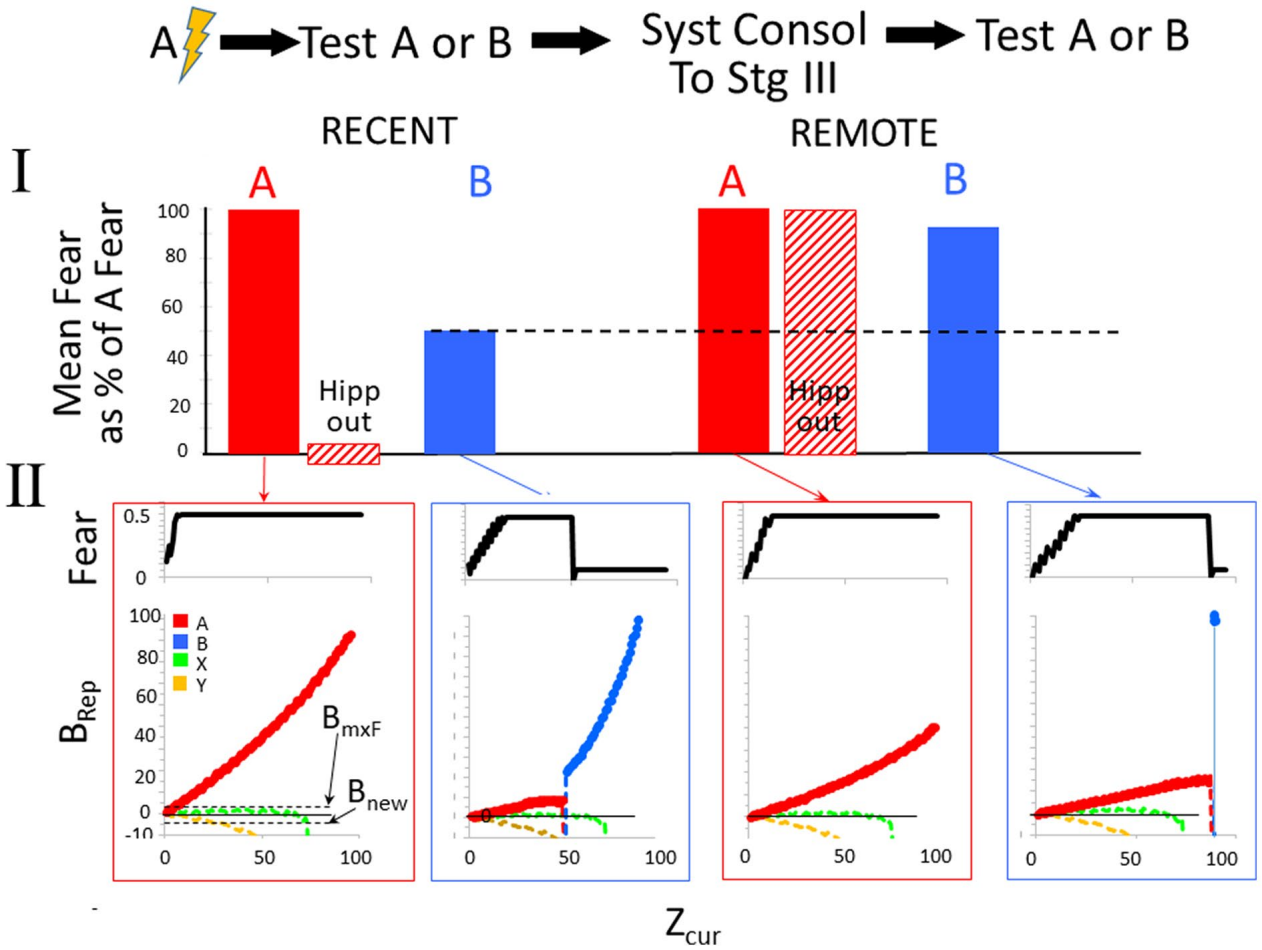
Remotely represented contexts hyper-generalize to unfamiliar contexts in the same category as the conditioned ones. Bacon was placed in a new context (context A) and conditioned when *Z_cur_* = 75. It was then tested for 95 intervals in either the conditioned context or a novel moderately similar one in the same category (*O_ptcAB_* = 0.6) either before or after allowing systems consolidation to proceed to Stage III. The hippocampus dependence of the conditioned response was also evaluated before and after systems consolidation. Only one kind of test was done per simulation. **(I)** Average fear across each test session and effect of hippocampal inactivation during the test session. **(II, Top graphs)**. Fear over the course of the session. **(II, Bottom graphs)**. Weight of evidence for contexts relevant to this experiment. The *B_Rep_* value for Rep A is plotted red and that of Rep B in blue. Bacon is also assumed to have representations for various established Stage III contexts. We assume that some of these are in the same category as Contexts A and B (Category X—we plot the *B_Rep_* value of a typical one of thee as a green line) and others in a different category (Category Y—we plot the *B_Rep_* value a typical one of these as a yellow line). The *B_rep_* curve for whichever representation is active is plotted as a bold line. The graph also indicates the value of *B_Rep_* at which maximal fear expression occurs (*B_mxF_*) and the value below which a known context must fall for a new representation to be created (*B_new_*).At the start of both the recent and remote generalization tests in Context B, Rep A, which is more similar to B than to either of the established contexts, has the highest *B_Rep_* value and is activated. The fear conditioned to it is therefore expressed in proportion to the *B_Rep_* value of the active representation. Thus, fear gradually increases at the start of both recent and remote test sessions in B. However, as more and more attributes of Context B are observed, the weight of evidence that Bacon is in Context B eventually stops growing and declines. When Rep A is recent and is associated with a substantial complement of attributes that are particular to Context A, this decline starts when *Z_cur_* is about 45, and *B_Rep_* rapidly becomes very negative. When it falls below *B_new_*, a representation of context B is created. Because Context B is fairly similar to A, its representation has some overlap with that of A and so some conditioned CA3 cells are conditioned ones; hence, a small amount of fear is now expressed. During the remote generalization session, the story is similar, except that attribute information distinguishing B and A is less than was the case when Rep A was recent, with the result that *Z_cur_* must reach much higher levels before *B_Rep_* of Rep A falls and fear is shut off.

Figure 9 shows a BaconREM simulation of an experiment in which A and B are in the same category. The particular attributes of A and B overlapped by 60%, and since they are in the same category, the categorical attributes overlap by 100%. As the bar graphs indicate, the results of the simulation are consistent with the above description of the common experimental finding (Table 2, Prediction E).

In the bottom half of the figure, graphs of fear and *B_Rep_* over the course of test sessions in contexts A and B are shown (explanations in the figure caption). During tests in B, when A’s creation and conditioning were recent, BaconREM, at first not knowing there was a context B, initially ‘thought’ it was in A (i.e., activated Rep A) and thus expressed fear, but about halfway through the session ‘realized’ this was not the case (i.e., *B_Rep_* of Rep A fell below *B_new_*) and a representation of context B was created. Once this happened, relatively little fear was expressed because B’s representation cells overlapped those of context A relatively little. In the remote case, the ‘realization’ that A was not B and the creation of Rep B occurred only at the very end of the session (i.e., when *Z_cur_* was almost equal to *N_atr_*); hence, high fear was expressed throughout almost the whole session. Thus, as in real animals, when tested using an unfamiliar context B, remote fear in BaconREM shows extreme generalization. A corollary of this is that false conditioning [conditioning to a familiar context when an individual is actually in a somewhat similar novel one (Rudy and Oreilly, 1999)] will be much more likely to occur to a remote than to a recently created representation (Table 2, Prediction F).

##### 3.2.2.2 Hyper-generalization and normal generalization during Stage II

**Figure 10:**
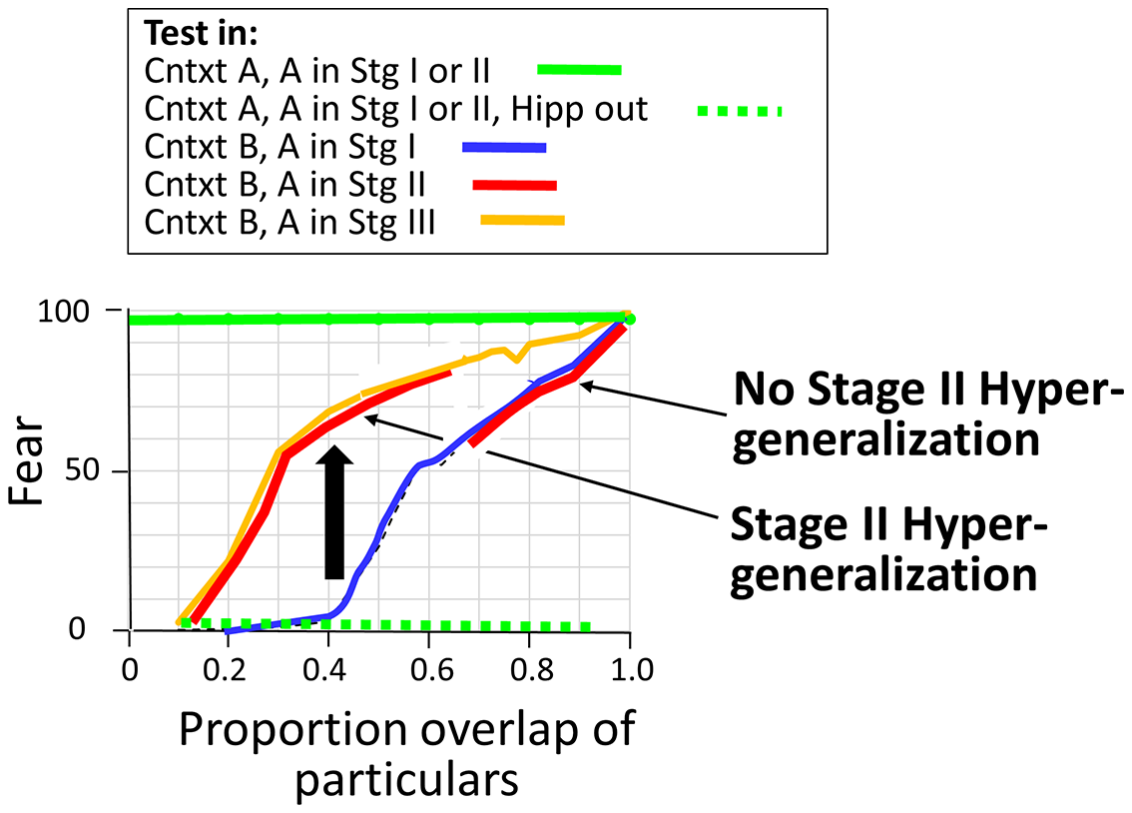
Generalization to unfamiliar contexts during Stage II of systems consolidation. During Stage II of the systems consolidation process, hippocampal and cortical versions of a context’s representation compete for activation. If the generalization of fear from a conditioned context A that is in Stage II to an unfamiliar context B is tested, the context A representation that will be activated prior to forming a representation of B depends on the degree of similarity of the two contexts. If they are fairly similar, the fear expression will be as it would be if the representation were in Stage I. However, if the contexts are not fairly similar, the fear expression will be as it would be if context A’s representation were in Stage III. The reason for this difference is that when the new context B is very similar to A, the *B_Rep_* of hipp Rep A is greater than that of cortical Rep A because A and B are similar, but *Z_rec_* of the Hippocampal rep is greater than that of the Cortical one, and hence the hippocampal rep is the one activated until Rep B is created. However, if new context B is substantially different from A, *B_Rep_* of Hippocampal Rep A is less than that of Cortical Rep A because context B is dissimilar to A, and the hippocampal representation has both a higher *Z_rec_* and more discrepant associated attributes than the cortical one. Note that irregularities in the above curves occur because the arguments of some functions used in the computations must be integers. The irregularities are due to the need to round some values to the nearest integer.

The above simulations compared generalization behavior during Stage I, when the hippocampal representation mediates behavior, to that during Stage III, when the cortical one does so. However, behavior during Stage II, when both the hippocampal and cortical versions of the conditioned representation are operational and compete for activation, is of interest because it makes a counter-intuitive prediction that deserves testing and because this prediction may account for some as yet unpublished data considered in the Discussion section. When Bacon is tested in a novel context that is similar to a conditioned context whose representation is in Stage II, either the hippocampal or cortical representation of the conditioned context will get activated until such time as a representation of the novel context is created. However, which one it will be depends on how similar the conditioned and novel contexts are. If they are fairly similar, then it will be the hippocampal version, whereas if they are quite different, it will be the cortical one. This is illustrated in Figure 10 and explained in its legend. As a result, when the conditioned representation is in Stage II, BaconREM generalizes as though it were in Stage I if the generalization test context is fairly similar to the conditioned one, whereas it generalizes as if it were in Stage III if the test and conditioning contexts are less similar. However, since fear has not yet transferred to the cortical representation, fear is hippocamus-dependent during Stage II no matter which representation is activated; in this case, the cortical representation evokes fear via the CA3 cells with which it remains connected permanently once Stage I is complete (Table 2, Prediction G).

##### 3.2.2.3 Experiments that should not show hyper-generalization of remote fear

There are several sorts of experiments, few of which have been done, in which remote fear in BaconREM would either fail to hyper-generalize or even do the reverse.

To the best of our knowledge, virtually all experiments so far done to test the generalization of remote context fear have used unfamiliar test stimuli and gotten hyper-generalization. However, according to the present model, this occurs because BaconREM, not having a representation of the test context, ‘thinks’ it is in the conditioned context and so expresses fear. If Bacon already had a representation of the unconditioned test context, it would activate that representation and express much less fear (Table 2, Prediction H). It would show slightly greater generalization when the representation of the feared context was remote, but hyper-generalization would not occur.

**Figure 11:**
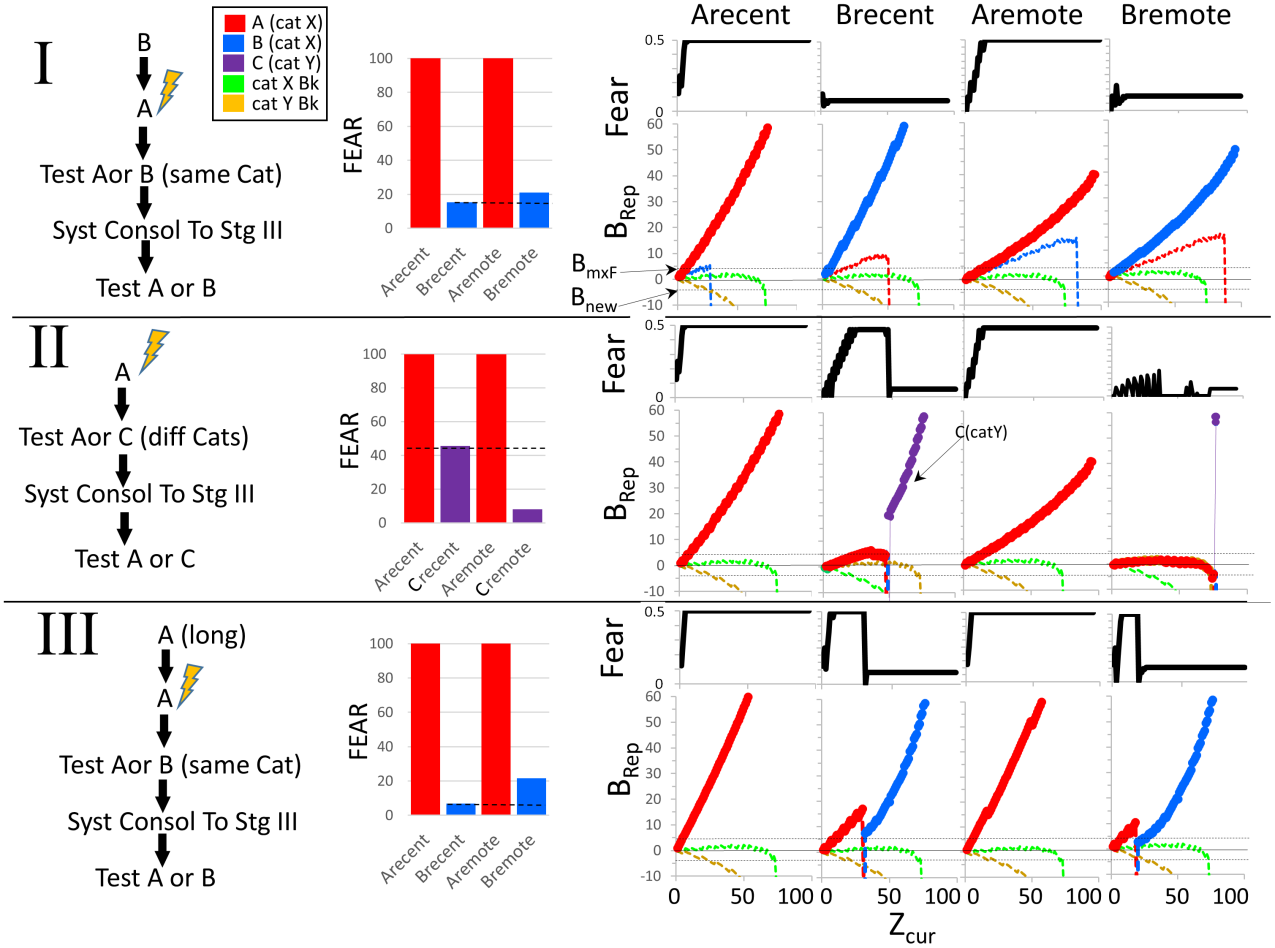
Lack of hyper-generalization of remote fear. **(I)** Hyper-generalization of remote fear fails to occur if the test context is familiar. If there is already a representation of the test context, BaconREM does not initially mis-identify the test context as the conditioned one but instead activates the test context’s own representation. Under these circumstances, there is somewhat more generalization when both contexts are remote than when both are recent because the generalization function (Figure 7) has a higher value for any given degree of attribute overlap. However, the extreme hyper-generalization seen when the test context has no representation of its own does not occur. **(II)** *Hyp*o-generalization of remote fear can occur if (unfamiliar) test contexts are in a different category from the conditioned one. In this simulation, the overlap of the particular attributes of contexts A and C (of different categories) was 0.75. As the bar graph shows, there was less generalization in the Remote than in the Recent case. In the Recent case, Rep A stopped being activated a little less than halfway through the session. In the Remote case, Rep A and the established representation that was in the same category competed for activation for about the first 3/4 of the session. However, because systems consolidation had caused additional categorical attributes that differentiated context A from C to get added to Rep A, *B_Rep_* was lower when Rep A was active in the Remote than in the Recent case. Thus, even when Rep A was active in the Remote case, *B_Rep_* was very low, and hence, fear expression was slight, as can be seen by comparing the first half of the Recent and Remote generalization tests. When, in both the Recent and Remote sessions, Rep C was created, fear became very low. **(III)** Hyper-generalization of remote fear to an unfamiliar context fails to occur if a great deal is known about the conditioned context. That is because, as discussed with respect to Figure 5, if the number of attributes associated with the hippocampal version of a representation is especially large, a greater proportion of them tends to get copied to the cortical version. This is illustrated in this experiment where BaconREM was pre-exposed to context A until *Z*_*cu*r_ = 98, so a great deal would be known about it. As the bar graph shows, there is slightly more generalization in the remote than in the recent case, but nothing as extreme as in Figure 9.

Figure 11I shows a simulation of this kind. In this experiment, BaconREM was pre-exposed to context B before the conditioning session in context A. The bar graphs are just as expected. Moreover, as can be seen from the graphs of fear and *B_Rep_* as a function of *Z_cur_*, there was, as expected, never a period of BaconREM ‘thinking’ it was in A, so Rep B (hippocampal in the recent case and cortical in the remote one) was activated from the very start of the test sessions. Because, for reasons explained in our discussion of generalization mechanisms, there tends to be somewhat more generalization when a pair of representations are both cortical than when at least one is hippocampal, there is a little more generalization in the remote than the recent case, but not to anywhere near the extent that is the case when context B is novel. Experiments need to be done to test this predicted difference between familiar and unfamiliar context B’s.

A case in which BaconREM does not predict greater generalization during remote tests even when the test contexts are novel occurs when a conditioned context A and a test context C are in different categories. When that is the case, generalization using novel test stimuli can actually be *less* when the tests are done after systems consolidation than before (Table 2, Prediction J). This is essentially because if the contexts are in different categories, the pre-known categorical attributes that get added to the representation of A when it becomes remote may *increase* the discrepancy between the attributes associated with contexts A and C. A simulation that illustrates this is shown in Figure 11II.

A final case in which BaconREM does not predict hyper-generalization occurs when especially substantial exposure to context A occurred at the start of the experiment before conditioning to allow an unusually great deal to be learned about that context (Table 2, Prediction K). Biedenkapp and Rudy (2007) found that when this was done, tests done after allowing time for systems consolidation failed to show hyper-generalization. A simulation of this experiment is shown in Figure 11III. Hyper-generalization fails to occur because the extra exposure to context A at the start of the experiment resulted in so much being learned about context A that a larger than normal fraction of particular attributes associated with the hippocampal (Stage I) representation were retained during systems consolidation, as discussed above with respect to Figure 5.

#### 3.2.3 Reminder experiment

A number of experiments have been done in which, after the development of systems consolidation (when animals were presumably at Stage III according to the present model), tests of generalization that would normally have shown a substantial loss of context specificity of fear were given a “reminder” exposure to the conditioned context before testing generalization. The effect of this was to restore specificity to the conditional stimulus *and* to make fear again hippocampus-dependent (e.g., Wiltgen and Silva, 2007; Winocur et al., 2009; Sekeres et al., 2019). Such results are usually taken to imply that the reminder improved access to information that was retained in the hippocampus even after fear became remote.

**Figure 12:**
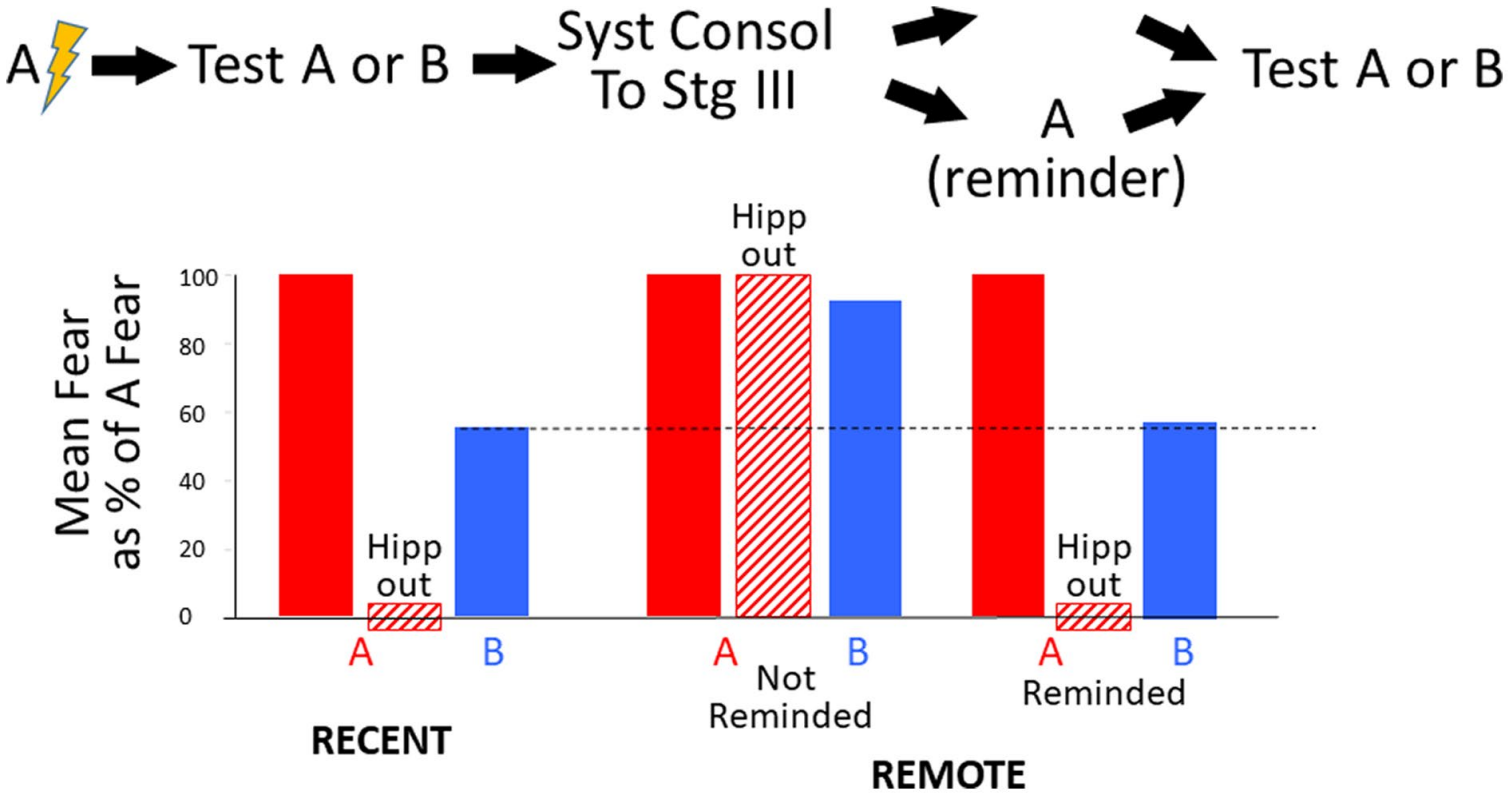
The ‘Reminder’ effect. Abolition of hyper-generalization due to a “reminder” exposure to a feared, remotely represented context. Context B is unfamiliar, and the conditions for this simulation were identical to those for the simulation of Figure 9. *Z_cur_* = 60 at the end of the reminder session.

Figure 12 simulates such an experiment. The “reminder” appears to have the same effect as in real animals. However, the reason is quite different from what has been speculated regarding the biological case. Bacon permanently loses information about contextual details stored in its hippocampal representation circuitry when a representation becomes remote. But the “reminder” causes additional attributes, either ones that had not been noticed before or ones that had previously been associated with the representation but forgotten, to be learned or relearned, and control is returned to the hippocampus to make possible a new round of systems consolidation to incorporate the new information into the cortical knowledge structure (Table 2, Prediction L). Note, however, that the reminder effect should only occur if exposures to the conditioned context are long enough to produce a *B_Rep_* high enough to allow updating (Table 2, Prediction M).

If the reminder effect really does depend on new learning, it would not be expected to occur if the Hebbian plasticity of relevant synapses were prevented. Therefore, we would expect that an NMDA receptor blocker would prevent reminders from abolishing post-consolidation hyper-generalization (Table 2, Prediction N). This is considered further in our Discussion section.

## 4 Discussion

The BaconREM model of remote context fear starts with Marr’s hypothesis on the role of the hippocampus in memory and adds several further ingredients:

(1) As does Marr’s theory and its various derivatives, BaconREM postulates that an event (or context) memory is initially formed by selecting a small set of hippocampal cells to represent it and then potentiating various circuit synapses so that, when even a modest fraction of the encoded event’s attributes are observed, they activate the full hippocampal representation which then causes a recollection of all known aspects of the event. However, the theory does not provide a way for the hippocampus to know whether a situation the animal is in is a new one that should cause encoding or an already known one that should cause recall but not necessarily new learning. BaconREM solves this problem by postulating that some region of the brain (perhaps the pre-frontal cortex) compares the attributes of what is currently being observed to those of its most relevant existing memory and computing the Bayesian Weight of Evidence that its current situation really is the same as the remembered one. Then, based on the outcome of this calculation, it may either form a new memory or associate newly observed attributes with the active representation (updating).
(2) It is widely believed that when hippocampal memories become remote, they move to the neocortex, where they become less detailed, more generic, and integrated into the cortex’s general knowledge structure. To incorporate something akin to a general knowledge structure into the model, we let contexts be categorized and have both generic (“categorical”) and context-specific (“particular”) attributes. When created, a representation is hippocampal and associated with whatever subset of contextual attributes it has so far noticed. But when it becomes cortical, it becomes associated with all those attributes of its category that are known from past experience, even though they have not been observed for this context. Additionally, only a limited number of observed attributes not known to be categorical do so. Thus, in this model, remote memories gain information from a sort of general knowledge structure but lose information about the particularities of a given context. We also postulate that whereas the construction of a cortical representation is a not terribly lengthy process, it is followed by a substantially lengthier process of variable duration, during which attribute information associated with the hippocampal representation is used to upgrade the cortex’s categorical knowledge structure.
  It appears that a number of cortical regions, including the medial prefrontal (and the richly associated thalamic nucleus reuniens), cingulate, and retrosplenial cortex, are all critically involved in systems consolidation and the recall of remote context fear memories (e.g., Frankland and Bontempi, 2005; Corcoran et al., 2011; Katche et al., 2013a,b; Todd and Bucci, 2015; Kitamura et al., 2017; de Sousa et al., 2019; Fournier et al., 2021). Of possibly special interest is the retrosplenial cortex, which is intimately connected with both cortical and hippocampal areas that appear to participate in systems consolidation and where manipulations during the period when systems consolidation is occurring can have significant effects on the process (Katche et al., 2013a,b; de Sousa et al., 2019). However, there is not yet a sufficient understanding of any of these areas’ exact roles for us to have tried to incorporate them explicitly into our model. Moreover, in trying to understand these roles, it should be borne in mind that insofar as the ideas incorporated in the BACON models are correct, cortical circuitry may not only play a role in mediating fear behavior but also a role in assessing memory validity as instantiated by *B_Rep_* in these models.
  It should be noted that the hippocampal independence of remote contextual fear (the so-called Standard Model) has recently been called into question by the finding that very sudden optogenetically produced inactivation of CA1 caused a loss of remote contextual fear (Goshen et al., 2011). In BaconREM, remote representations and the best available hippocampal one compete for activation in each computational cycle. The circuitry mediating this competition is unknown. But it would not be surprising if the sudden vanishing of the hippocampal representation from the set of competing representations were to cause this circuitry to malfunction and fail to activate the best available remote representation (in this case, the fear-conditioned one). Until there is a fuller understanding of the basis for the Goshen et al. effect, we have chosen to stick with the standard model in designing BaconREM. It should also be noted that Goshen et al. found that the same optogenetic stimulation did not disrupt remote memory if it started slightly before testing, suggesting that it is the sudden disruption of hippocampal function that briefly interfered with remote memory recall.
(3) Finally, since we believe that information associated with the hippocampal representation is needed for systems consolidation to occur, our hippocampal representations remain intact until a consolidation period is completed.

The model makes a number of predictions that correspond to known phenomenology, as well as a number that have not been tested, as listed in Table 2. However, we focus this discussion on (i) the fundamental question of whether the hippocampus continues to be the repository of some aspects of contextual fear memories even after they become remote, (ii) some important differences between recent and remote representations, and (iii) our conception of how the systems consolidation process is organized.

### 4.1 Dual memory vs. time-limited theories of systems consolidation

Following from initial ideas and observations of Marr (1971) and Scoville and Milner (1957), it was thought that rapidly established memories are initially laid down in hippocampus-centered circuitry but later transferred to primarily cortical circuitry for potentially permanent storage with good retention of aspects of the events that are shared with other similar events but much poorer retention of details that are specific to the particular experience (“time-limited” theory of hippocampus-centered memory). However, later consideration of both human and animal data (e.g., McClelland et al., 1995; Nadel and Moscovitch, 1997; Wiltgen and Silva, 2007; Winocur et al., 2007; Wang et al., 2009; Wiltgen et al., 2010; Nadel et al., 2012; Wiltgen and Tanaka, 2013; McClelland et al., 2020) has led to the alternative hypothesis that when memories that are initially hippocampus-based become remote, context/situation-specific details *remain* permanently stored within the hippocampus while attributes that the context/situation shares with other similar situations become stored cortically—dual memory (hippocampus + neocortex) theories.” However, it seems to us that if situational detail were to be permanently stored within hippocampal circuitry, then, given the way this circuitry is thought to operate, there would begin to be crosstalk between memories as more and more of them are accumulated in hippocampus-centered circuitry.

One of the most important findings leading to dual memory theory has come from experiments on the effects of reminder exposures to a feared, remotely remembered context: The effect of such exposures is to cause a cessation of the hyper-generalization usually found for remote context fear memories and a return to hippocampal dependence of the conditioned fear (e.g., Wiltgen and Silva, 2007; Winocur et al., 2009). This is commonly taken to imply that memory of details that permitted differentiation of the conditioned context from a similar alternative context was stored permanently in the hippocampus, and the reminder exposure somehow made it again accessible. The present model generates similar results (Figure 12, Table 2, Prediction L) because the reminder exposure causes the learning of contextual details (or the relearning of details that were discarded during the systems consolidation process) that then makes possible more precise identification of the conditioned context. Moreover, the return to hippocampal dependence occurs because the hippocampus is required for the systems consolidation process that incorporates the newly acquired information into the cortical knowledge structure.

It seems to us that the crucial question at issue here is whether (1) the hippocampus and cortex store different versions or different aspects of remote memories, both of which are permanent (dual memory theories) or (2) whether hippocampal memories are always time-limited and the permanent form of contextual memories is entirely non-hippocampal (consolidation or time-limited theories). It does not appear to us that at present there are experimental findings that can settle this matter. The finding that even in the absence of a reminder exposure to a remotely remembered conditioned context, placement in that context causes more hippocampal cFos activity than does placement in a similar novel context (Sekeres et al., 2019) at first seemed to us to strongly favor a dual memory theory over the present approach. On the face of it, this suggests that the hippocampus retains a memory of the context even after the context’s representation becomes cortical. However, we then realized that this is not inconsistent with the present model because the model postulates that cortical representation cells retain permanent connections with the CA3 cells of their hippocampal representation progenitor so as to make possible generalization of fear between recent and remotely represented contexts. Such connections could well be the basis for the results of Sekeres et al.

In the absence of definitive evidence for one type of theory over the other, we would favor the present sort of theory because we think it is more compatible with Marr’s theory of the hippocampus, which seems to us to be plausible, to have considerable evidence in favor of it, and to be rather widely accepted. There is, however, one kind of experiment that we think would go to the heart of the matter. The crucial distinction between the two types of theory is that dual memory theory proposes that reminder experiments restore context specificity by promoting access to permanently established hippocampal detail memory, whereas the sort of theory embodied in BaconREM proposes that reminders cause *new* (i.e., hippocampal) learning (or relearning) of details that upgrade specificity and must return control to the hippocampus because it plays an essential role in revising input to the cortical version of its representation and the cortical knowledge structure generally. Therefore, prevention of new learning should not interfere with the return of contextual specificity of remote fear (i.e., abolish hyper-generalization) under dual memory theories but should do so if real animals behave like BaconREM (Table 2, Prediction N).

New learning could be prevented by applying either NMDA or protein synthesis inhibitors during the reminder session. However, if dual memory theories are correct, reminder sessions might cause the recall of the “permanent” associations that are hypothesized to be present in the hippocampus and thereby lead to a need for a period of protein synthesis to re-stabilize them (i.e., to a need for cellular memory “reconsolidation”). Thus, protein synthesis inhibitors might prevent the restoration of contextual specificity even though no new learning was occurring and, therefore, would not distinguish between the two kinds of theory. Hence, the proposed experiments should utilize NMDA receptor inhibitors.

In fact, hippocampal protein synthesis inhibitors applied during re-exposures simply abolish fear, which never returns, whereas we believe that dual memory theories would predict that hyper-generalizable fear would continue to be elicited by cortical circuits. However, under a BaconREM-type theory, it might well be that control of behavior has been returned to the hippocampus by the updating process, and a loss of hippocampal associations due to reconsolidation would result in a permanent loss of fear.

### 4.2 On new learning during recall

Exposure to a context that is familiar or reminiscent of one that is will cause activation of the context’s representation and thereby a memory of all that is known about the context. If observed features of the context differ somewhat from those recalled, new learning may occur. If we understand them correctly, some current theories (e.g., Nadel and Moscovitch, 1997; Yassa and Reagh, 2013) postulate that each time a situation is recalled, a new memory is formed that incorporates the attributes observed on that particular occasion. However, the BACON models make a somewhat more nuanced prediction: If the context that provoked recall differs sufficiently from the recalled context so that *B_Rep_ < B_new_*, then a new contextual representation is indeed created. However, if the current and recalled contexts are sufficiently similar so that *B_Rep_ > B_add_*, then BaconREM assumes that the observed features of the current context that are not familiar were simply not noticed previously or had been forgotten, so it now associates those attributes with the *existing* representation (what we have referred to as “updating”), while if *B_Rep_* is neither sufficiently less than *B_new_* nor sufficiently greater than *B_add_*, no new learning at all occurs. One easily testable consequence of this prediction is that recall should cause a return of hippocampus-dependence of conditioned fear and a cessation of hyper-generalization only if a contextual exposure is long enough to allow a subject to make an informed decision as to whether the current context is new (or altered) or is the one already known (Table 2, Prediction M).

### 4.3 Some consequences of the special properties of remote representations

When contextual representations become remote in BaconREM, they lose associations to context-specific (particular) attributes and gain associations to known attributes of the context’s category. As a consequence, when placed in a new context of a familiar type (i.e., category), they are initially likely to ‘think’ that they are in an already known, remotely represented context of that type. This has a number of implications.

#### 4.3.1 Difficulty of making new representations if there are already remote representations in the same category

As seen in the simulations of Figure 8, if the category of a newly encountered context is one that BaconREM knows, a representation of the context will only form if BaconREM is able to observe substantially more about the context than would be necessary if the category were unfamiliar. Thus, representations specific to a novel context are relatively unlikely to be made (or would require especially long exposures to the new context) if the context’s category is already known than if it is not.

Whether there are, in reality, contexts for which individuals do not have pre-existing known categories is an interesting question. The categorical structure is presumably something a real individual would develop by noting commonalities of attributes among those contexts with which it had become familiar, and categories might be subject to change as a function of what kinds of contexts the individual encountered over time. Perhaps real individuals always place contexts or situations they encounter into some category they have previously recognized, and our consideration of contexts for which BaconX has no category is unrealistic. Since, as indicated in Figure 8, the immediate shock deficit should be especially short in unfamiliar contexts for which an individual has no category, it would be interesting to see whether animals would become conditionable especially soon if conditioning were attempted in a highly unusual sort of new context.

#### 4.3.2 Generalization

It is widely believed that remote memories lose detail and become gist-like, and as discussed extensively in the Results section, it is consistent with this that when fear is conditioned to a novel context, the fear at first is relatively specific to the conditioned context, but after the context’s representation becomes remote, unfamiliar contexts that are similar evoke almost as much fear as the conditioned context (our “hyper-generalization”). BaconREM emulates this finding because when it is first placed in an unfamiliar context, it activates the representation of the most similar known context, which, if conditioned, evokes fear. If the representation is hippocampal, it is soon rejected due to *B_Rep_* soon going negative, and a new, unfeared representation is created, whereas if the representation of the known context is remote, it takes much longer to be rejected so that fear persists, resulting in hyper-generalization. *However*, BaconREM does not show hyper-generalization if the test context used is already known rather than novel (Table 2, Prediction H); in that case, Bacon activates the representation of the already known similar but unfeared context as soon as the context is entered. As far as we know, results for this sort of experiment have never been reported. They obviously should be tried.

#### 4.3.3 Extinction and matters relevant to exposure therapy for fear disorders

We have not included extinction mechanisms in BaconREM because it would have complicated the things we wished to focus on. However, extinction would be added to BaconREM in exactly the same way that it was added to BACON to create BaconX, a model of extinction for recent fear memories (Krasne et al., 2021), and it is worth pointing out a few ways that extinction of remote fear would differ from that of recent fear if extinction mechanisms were added to BaconREM in this way.

Extinction occurs in BaconX as the result of fear inhibition becoming conditioned to contextual representations when fear responses are non-reinforced. However, such conditioned inhibition only becomes permanent (carries over from one session to another) if, at the end of the session during which non-reinforcement occurred, the individual is very confident that it really is in the feared context, i.e., if *B*_*Re*p_ is greater than a value we call *B_xBtwn_* (“*xBtwn*” for “Between session extinction”; Zinn et al., 2020; Krasne et al., 2021).

Given these assumptions, when extinction is done in the context of conditioning, persistent extinction of fear is more difficult to get when the conditioned representation has become remote because *B_Rep_* generally rises more slowly for remote than for recent contextual representations (Figure 9B), and thus it takes longer for it to reach *B_xBtwn_* or it might not be reached at all.

**Figure 13:**
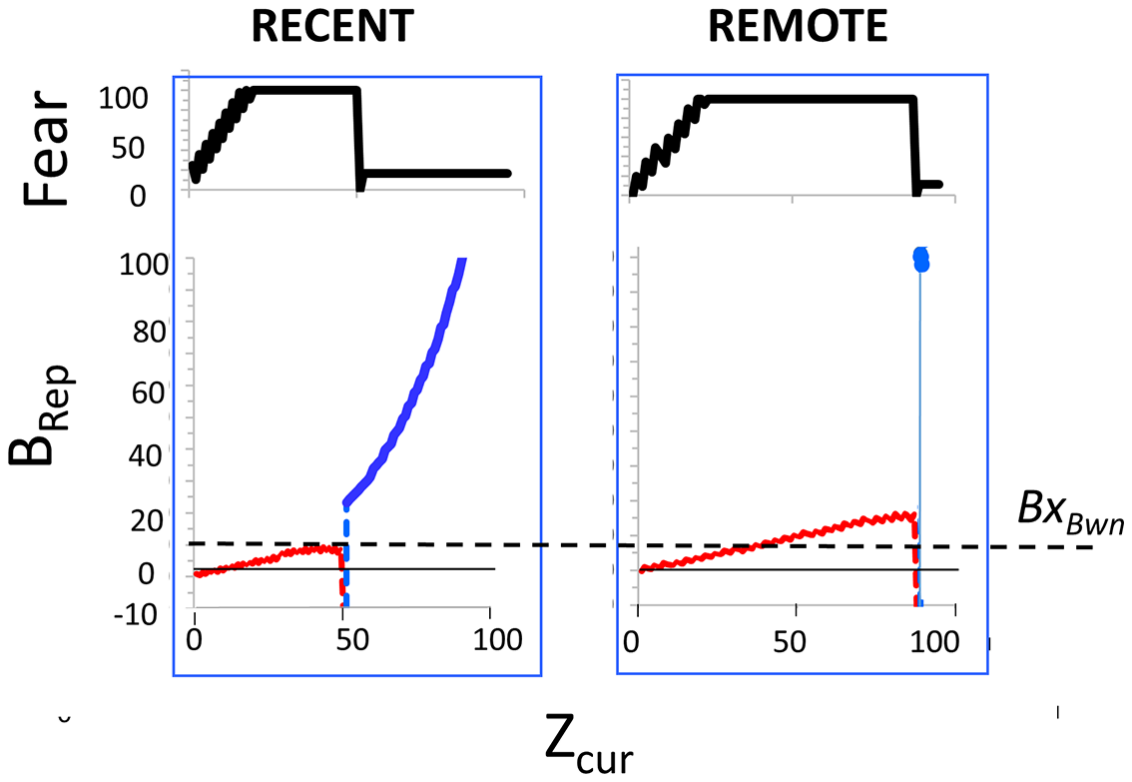
Exposure therapy is a situation when conditioned fear is recent vs. remote. These graphs show what fear and *B_Rep_* would be like during a mock virtual reality extinction session in which the proportion of overlap of the context’s particulars with those of the conditioning context was 0.6 and the category known, as in the simulations of Figure 9. Fear inhibition would become conditioned to the conditioned context’s representation while that representation was active and would persist beyond the session to the extent that the *B*_Rep_ of the (red-graphed) representation was greater than at the end of the session. When the conditioned representation was recent, any inhibition that became conditioned to the feared representation would not outlast the session even if the session were terminated before a representation of the therapy session itself was created because *B_Rep_* of the conditioned context never got above *B_xBtwn_*; however, when the representation was remote, termination of the session a bit before *Z_cur_* = 80 would result in some long-lasting inhibition that would be effective in suppressing fear in real-world contexts similar to the conditioning contexts because *B_Rep_* of the conditioned context did exceed *B_xBtwn_* in the latter part of the session.

However, when extinction is carried out in contexts similar to *but not the same as* the conditioning context, as would be done in virtual reality exposure therapy sessions, the situation is rather different. As discussed extensively for BaconX (Krasne et al., 2021), for the conditioned inhibition produced by extinction in the therapy situation to be truly effective in suppressing fear in the real world, it must become conditioned to the conditioned context itself. But if contextual fear was recently acquired, then the patient is likely to form a representation of the therapy context by the time that *B_Rep_* has become great enough that any inhibition conditioned to the conditioned context could become permanent (i.e., before *B_Rep_* of the conditioned context reaches *B_xBtwn_ –* see Figure 13-RECENT), and consequently any extinction that occurred during the session would be mostly due to inhibition conditioned to the therapy session context rather than the conditioned context itself. Therefore, in a BaconREM-like individual, extinction of recently acquired fear would not be likely to transfer well to the real world. However, once the representation of a feared context becomes remote, exposure to a virtual reality replica of it before the development of a representation of the therapy context will activate the representation of the feared context itself, produce a high enough *B_Rep_* value to cause persisting extinction (i.e., *B_Rep_* > *B_xBtwn_*), and generate enough fear to cause inhibition to become conditioned to the conditioned context itself (see Figure 13-REMOTE). Therefore, so long as each therapy session terminates before a representation of the therapy context is formed, inhibition that will be effective in suppressing conditioned fear in any similar situation will be learned over a series of sufficiently short sessions.

### 4.4 The possibility of a stage of systems consolidation during which the cortical categorical knowledge structure is being revised and hippocampal and cortical representations co-exist (our Stage II)

As explained in the Results section, new representations in BaconREM are hippocampal, but soon after their creation, cortical versions of them are constructed (during our Stage I). A period of variable and possibly rather protracted duration follows during which attribute information associated with the hippocampal and cortical versions is used to try to upgrade the individual’s categorical knowledge (our Stage II). When this upgrading process has gone as far as it can with the available hippocampal and cortical information, any fear that is associated with the hippocampal version is transferred to the cortical one, the hippocampal version is taken out of operation, and its associations are either erased or become subject to being overwritten so that it does not interfere with the learning and recall of newly encountered contexts. The individual now always uses its cortical version (our Stage III).

There is at present no published evidence for the existence of our postulated period after representation creation during which hippocampal and cortical versions of representation co-exist and compete for activation (our Stage II). However, a rather counter-intuitive prediction made by our model, illustrated in Figure 10 and explained in its legend, is that if generalization of fear to novel contexts were tested during Stage II, one would find that test contexts that are fairly similar to the conditioned context whose representation was in Stage II would activate the hippocampal version and fear would generalize as it does during Stage I whereas less similar test contexts would activate the cortical version, and fear would hyper-generalize as it does during Stage III. This prediction should be easy to test. A hint that it might be correct comes from as yet unpublished experiments on an animal model of PTSD (SEFL — Rau and Fanselow, 2009; Perusini et al., 2016; Rajbhandari et al., 2018) in which hyper-generalization did appear to develop quite early, well before hippocampus-independence would be expected (i.e., as in our Stage II when testing generalization to a rather dissimilar context; Fanselow, unpublished). In these experiments, for reasons related to the investigators’ particular objectives, an effort was in fact made to make the test contexts quite different from the conditioning ones. Moreover, because this was a model of PTSD, the conditioning procedure used was unusually stressful and thus may well have had contextual features that enhanced its difference from the context used to test generalization. This data when published will provide evidence that hyper-generalization and hippocampal independence are not always tied together and will tend to validate the existence of a Stage II-like state, but experiments explicitly examining the effect of context similarity at times when a representation would be likely to be in our Stage II would still be needed to evaluate the predictions of Figure 10. It should be said, however, that if it should turn out that cortical representations do not become functional until hippocampus-independence develops at the end of the systems consolidation process and hyper-generalization does not in fact occur until then, some revisions of our model would be required, but not any of its central features.

## 5 Conclusion

BaconREM generates a number of predictions (Table 2), some of which are supported by existing data and, we believe, none contradicted by it. The as-yet-untested ones deserve testing. However, we think that the most important thing to have come out of this modeling effort is the recognition of the possibility that context fear conditioning characteristics that have been thought to provide compelling evidence for the dual trace theory of context fear memory may in fact be explained by the more traditional “standard model,” in which the memories of contextual attributes that are formed within the hippocampus and its connections to the cortex are transitory and become subject to erasure once a cortical version of the representation gets constructed and becomes fully operational.

## Data availability statement

The original contributions presented in the study are included in the article/supplementary material, further inquiries can be directed to the corresponding author.

## Author contributions

FK: Conceptualization, Writing – original draft, Writing – review & editing. MF: Conceptualization, Writing – review & editing.
